# Quantum weak value amplified terahertz chiroptical measurement

**DOI:** 10.1515/nanoph-2024-0685

**Published:** 2025-04-16

**Authors:** Liping Xu, Jiangtao Xu, Xin Yao, Rumin Zhang, Gang Wen, Lei Wang, Xingxing Lu, Zaoxia Li, Wenquan Liu, Dongshan Wei, Xiaoli Li, Tianying Chang, Hong-Liang Cui

**Affiliations:** The College of Nuclear Technology and Automation Engineering, Chengdu University of Technology, Chengdu, Sichuan 610059, China; Shenzhen Institutes of Advanced Technology, Chinese Academy of Sciences, Shenzhen, Guangdong 518055, China

**Keywords:** THz, phase, amplitude, chirality, optical activity, weak-value amplification

## Abstract

A precise method for phase and amplitude detection in both the time and frequency domains of terahertz spectroscopy based on the weak-value amplification technique is proposed and demonstrated. Within the weak-value amplification scheme, the imaginary weak value enhances variations in the terahertz phase signals, whereas the real weak value amplifies changes in the terahertz amplitude signals. By employing various postselections in the terahertz weak measurement procedure in detecting minute changes of the phase and amplitude of the terahertz wave, we achieved a phase change range from −0.0187 rad to 0.0183 rad with an interval of 0.004 rad and an amplitude change range from −0.0238 rad to 0.0228 rad with an interval of 0.0056 rad. This results in a phase and amplitude measurement resolution of 10^−4^ rad in the time domain. In the frequency domain, $\left|E\right|$ spectra are calculated to assess phase and amplitude variations with respect to frequency or wavelength. We apply these methods to chiral detection, particularly in measuring optical activity such as circular dichroism (CD) and optical rotatory dispersion (ORD). Despite challenges such as strong terahertz wave absorption in aqueous solutions and weak optical responses from natural chiral materials in the terahertz band, we successfully conducted chiroptical spectroscopy on a relatively large volume (2.3 mL) of liquid (R)- and (S)-limonene, as well as lactose tablets with varying mass fractions. Furthermore, the carrier-envelope phase (CEP) shift, defined for one- or few-cycle time-domain terahertz pulses, was effectively achieved through the manipulation of a pair of terahertz polarizers in the terahertz beam path. Notably, when *ϕ*_CEP_ = 0, a 150 % increase in the absorption coefficient of lactose was observed when weak measurement techniques were employed, compared to conditions without such measurements. This effort yielded THz-ORD and THz-CD spectra, demonstrating the potential of our methods to overcome traditional limitations and provide new insights into the optical response, dynamic properties, and low-frequency vibrational modes of biomolecules and materials in low-energy states, ultimately facilitating the identification of chiral stereoisomers.

## 1 Introduction

Terahertz waves (THz) refer to electromagnetic waves with frequencies ranging from 0.1 to 10 THz, bridging the gap between microwave and infrared radiation [1], [2]. THz-based detection technology shows great potential due to its distinctive physical traits, including fingerprint spectral specificity, water sensitivity, nonionization, broadband, strong coherence, and low photon energy, setting it apart from other bands of the electromagnetic wave spectrum [3], whereas, as with other electromagnetic wave based spectroscopies, THz detections are typically characterized by the electric field vectors in complex representations of both amplitude and phase. Polarization is also a fundamental characteristic of electromagnetic waves, describing the vector nature of their electric field oscillations. The relative variations in the phase and amplitude of polarized THz wave basis vectors arise from distinct physical mechanisms: phase differences occur due to variations in the propagation velocities of the eigenvectors influenced by the medium’s refractive index, while amplitude variations result from deviations in the absorption coefficients due to the medium’s differential attenuation characteristics [4]. Flexible manipulations or estimations of the physical properties (amplitude and phase) of the THz polarization state provide key support for THz application systems in multichannel communication, beam tracking and radar detection, polarization imaging, and chiral detection of biomolecules, among others [1], [5], [6].

Optical chiral detection, probing the optical chiral response of molecules interacting with left- and right-circularly polarized waves, is primarily manifested as CD and ORD [7], [8]. This response elucidates the mechanisms of ORD and CD, which correspond to the phase and amplitude variations of the incident light, respectively [1], [4], [9]. These techniques are widely used to analyze biomacromolecules, such as proteins and nucleic acids, in the ultraviolet and visible light regions (200–800 nm). In contrast, vibrational optical activity, which includes vibrational circular dichroism and vibrational optical rotation, is observed in the infrared and far-infrared regions (1,000–100,000 nm). This method is utilized for analyzing molecular structure and vibration modes. Analyzing low-frequency vibrations and rotational characteristics at THz wavelengths poses significant challenges. The THz wavelength greatly exceeds that of typical chiral molecular structures, leading to weaker chirality of natural materials in this regime, which complicates the study of chiral properties in biomolecules and low-energy materials [10], [11], [12]. Additionally, strong absorption of THz waves by water molecules further impairs the precision and sensitivity of traditional THz spectroscopy in aqueous solutions. Recently, THz artificial microstructure devices (e.g., metasurfaces, subwavelength gratings, and photonic liquid crystals) have emerged, introducing geometric symmetry breaking to enhance THz chiral responses efficiently [1]. The chiral effect induced by these artificial structures surpasses that of natural materials by several orders of magnitude. Unlike metamaterials and metasurfaces, which have fixed chiral responses upon fabrication, photonic liquid crystals offer dynamic tunability through thermal, optical, electric, or magnetic field control [1], [13]. However, metamaterials, metasurfaces, and liquid crystals primarily focus on maximizing phase change, with some capable of achieving changes from −150° to 145° [1], [13], [14]. In summary, due to the strong absorption of terahertz waves in aqueous solutions and the weak optical response of natural chiral materials in the terahertz band, particularly concerning optical chiral responses, achieving the measurement of liquid natural chiral materials requires detecting unusually small changes in phase and amplitude, which ultimately facilitates the identification of chiral stereoisomers.

The advent of weak-value amplification (WVA) represents a transformative breakthrough in overcoming these challenges. Initially proposed by Aharonov, Albert, and Vaidman in 1988, WVA has emerged as a powerful tool in high-precision metrology [15]. Its unique ability to enhance signal variations while mitigating technical noise has made WVA indispensable for the precise measurement of subtle parameters [16], [17], [18], [19], [20], [21], [22], [23], [24], [25], [26], [27], [28], [29], [30], [31]. This remarkable amplification capability extends to diverse applications, including polarization rotations [32], [33], optical phases [32], [34], [35], chemical reaction rate [36], and more.

In this study, we developed a method that integrates THz systems with quantum weak measurements. Experimentally, we simultaneously estimated phase and amplitude variations in both time and frequency domain. Our findings demonstrate that the imaginary weak value is responsible for amplifying the THz wave phase shifts, enhancing our ability to detect minute changes in the propagation velocities of the eigenvectors. On the other hand, the real weak value amplifies the THz waves amplitude variations, allowing for precise measurement of differential absorption coefficients. Employing this approach, we successfully conducted chiroptical spectroscopy on liquid (R)- and (S)-limonene and lactose tablets with different mass fractions, yielding THz-ORD and THz-CD spectra crucial for chiral detection. These experimental outcomes underscore the system’s exceptional accuracy and practical feasibility.

## 2 Theoretical model

Creating a theoretical framework for establishing THz wave phase and amplitude based on WVA begins with preselecting the initial THz photon polarization state in a superposition of eigenstates

(1) $$\begin{array}{c} \left|\psi_{\text{pre}}\right\rangle =\frac{1}{\sqrt{2}}\left(\left|H\right\rangle +\left|V\right\rangle \right), \end{array}$$

where $\left|H\right\rangle$ and $\left|V\right\rangle$ denote the horizontal and vertical polarization eigenstates, respectively.

Small variations in the phase *α* and amplitude *β* are encapsulated by the interaction/evolution operator

(2) $$\begin{array}{c} {\hat{U}}_{\left(\alpha \right)}=\exp\left(\frac{i\alpha }{2}\hat{A}\right), \end{array}$$

(3) $$\begin{array}{c} {\hat{U}}_{\left(\beta \right)}=\exp\left(\frac{\beta }{2}\hat{A}\right), \end{array}$$

where $\left|H\right\rangle$ and $\left|V\right\rangle$ are the eigenvectors of photon polarization operator $\hat{A}=\left|H\right\rangle \left\langle H\right|-\left|V\right\rangle \left\langle V\right|$ , which denotes the observable of the system, i.e., the THz photon polarization in the present case.

To estimate the phase variation, the postselection state is

(4) $$\begin{array}{c} \left|\psi_{\text{post}\left(\alpha \right)}^{\pm }\right\rangle =\frac{1}{\sqrt{2}}\left[\exp\left(\mp i\kappa \right)\left|H\right\rangle -\exp\left(\pm i\kappa \right)\left|V\right\rangle \right], \end{array}$$

± represents the two symmetric (with respect to the exact perpendicular direction of the preselected polarization direction) postselection states.

According to the general weak value expression [34], the imaginary weak value is derived from the preselected and postselected states

(5) $$\begin{array}{c} A_{w\left(\alpha \right)}^{\pm }=\frac{\left\langle \psi_{\text{post}\left(\alpha \right)}^{\pm }\left|\hat{A}\right|\psi_{\text{pre}}\right\rangle }{\left\langle \psi_{\text{post}\left(\alpha \right)}^{\pm }\left|\right.\;\psi_{\text{pre}}\right\rangle }=\mp i\cot\kappa . \end{array}$$

The intensity of the THz wave after postselection corresponding to the state is given by

$$I_{\alpha }^{\pm }=I_{0}{\left|\left\langle \psi_{\text{post}\left(\alpha \right)}^{\pm }\left|{\hat{U}}_{\left(\alpha \right)}\right|\psi_{\text{pre}}\right\rangle \right|}^{2}$$

$$\;\approx I_{0}{\left|\left\langle \psi_{\text{post}\left(\alpha \right)}^{\pm }\left|\right.\psi_{\text{pre}}\right\rangle \right|}^{2}\left[1-\alpha Im\left(A_{w}\right)\right]$$

(6) $$\begin{array}{c} \;=I_{0}\text{si}{\text{n}}^{2}\kappa \left[1-\alpha \text{Im}\left(A_{w\left(\alpha \right)}^{\pm }\right)\right], \end{array}$$

where *I*_0_ denotes the initial THz spectrum intensity prior to postselection. The approximation in eq. (6) is valid when ${\left|A_{w\left(\alpha \right)}^{\pm }\right|}^{2}\left(\alpha^{2}\right)/4\ll 1$ .

To estimate the amplitude variation, the postselection state is

(7) $$\begin{array}{c} \left|\psi_{\text{post}\left(\beta \right)}^{\pm }\right\rangle =\cos\left(\frac{\pi }{4}\mp \chi \right)\left|H\right\rangle -\sin\left(\frac{\pi }{4}\mp \chi \right)\left|V\right\rangle , \end{array}$$

and according to the weak value expression, the real weak value is derived from the preselected and postselected states

(8) $$\begin{array}{c} A_{w\left(\beta \right)}^{\pm }=\frac{\left\langle \psi_{\text{post}\left(\beta \right)}^{\pm }\left|\hat{A}\right|\psi_{\text{pre}}\right\rangle }{\left\langle \psi_{\text{post}\left(\beta \right)}^{\pm }\left|\right.\psi_{\text{pre}}\right\rangle }=\pm \cot\chi . \end{array}$$

The intensity of the THz wave after postselection corresponding to the state is given by

$$I_{\beta }^{\pm }=I_{0}{\left|\left\langle \psi_{\text{post}\left(\beta \right)}^{\pm }\left|{\hat{U}}_{\left(\beta \right)}\right|\psi_{\text{pre}}\right\rangle \right|}^{2}$$

$$\;\approx I_{0}{\left|\left\langle \psi_{\text{post}\left(\beta \right)}^{\pm }\left|\right.\psi_{\text{pre}}\right\rangle \right|}^{2}\left[1+\beta \text{Re}\left(A_{w\left(\beta \right)}^{\pm }\right)\right]$$

(9) $$\begin{array}{c} \;=I_{0}\text{si}{\text{n}}^{2}\chi \left[1+\beta \text{Re}\left(A_{w\left(\beta \right)}^{\pm }\right)\right], \end{array}$$

where, *I*_0_ denotes the initial THz spectrum intensity before postselection. In eq. (9), the approximation is based on the condition that ${\left|A_{w\left(\beta \right)}^{\pm }\right|}^{2}\left(\beta^{2}\right)/4\ll 1$ . From eq. (9), it is seen that the postselected THz wave intensity diminishes according to the probability of selection sin^2^ *χ*, with the intensity alteration being proportional to the weak value.

## 3 Results and discussion

**Figure 1:**
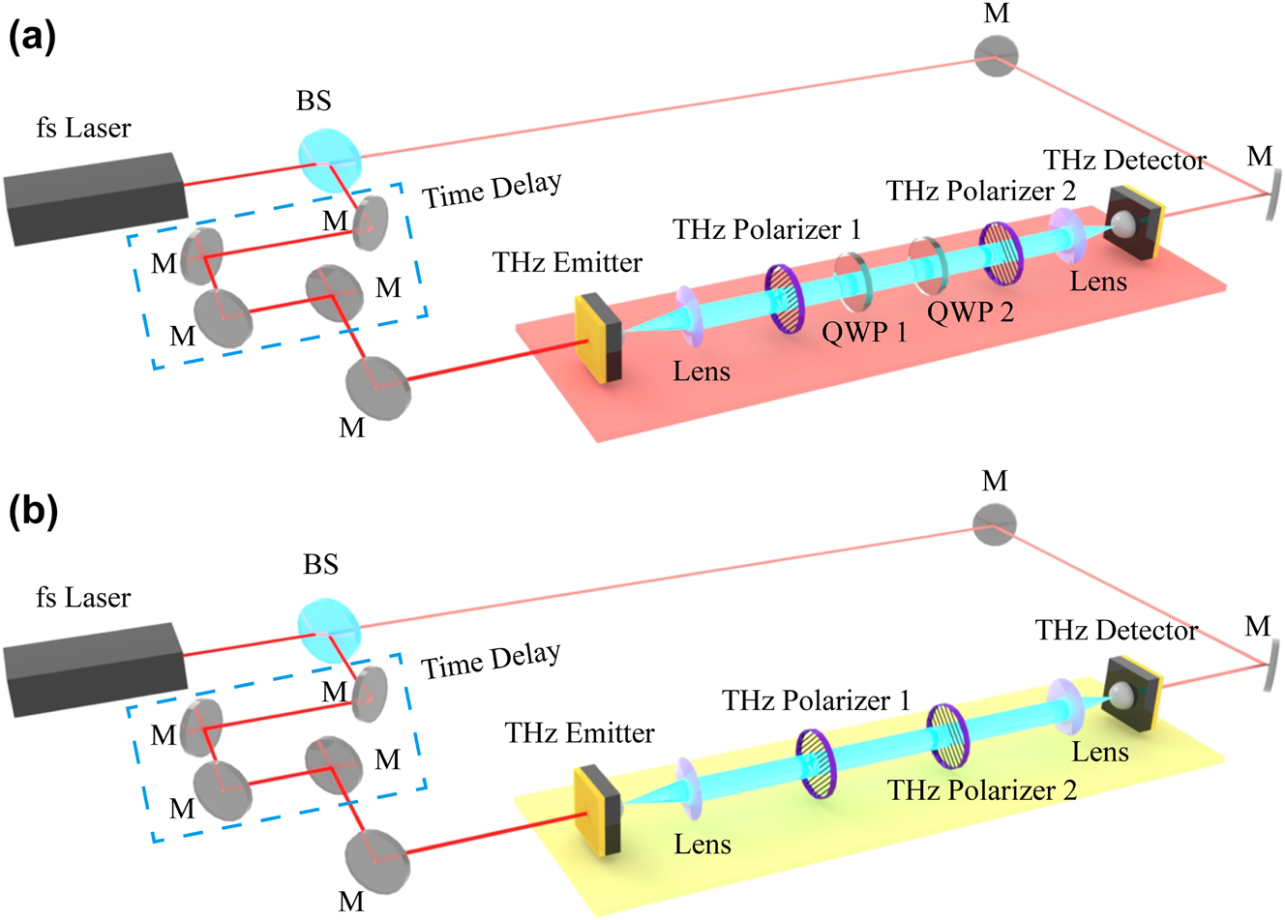
A schematic of the THz system based on the WVA approach. (a) Phase variation measurement system. (b) Amplitude variation measurement system.

A simplified schematic of the THz setup utilizing weak-value amplification to capture phase and amplitude variation data in a transmission configuration is illustrated in Figure 1. The THz emitter and detector are horizontal polarization and copolarized. Figure 1a depicts the WVA segment highlighted in color along the entire optical path. Initially, the THz wave passes through the first THz polarizer 1 (Product model: PWO0S-012-050, THz wire grid polarizers, wavelength range above 150 μm, extinction ratio >1,000:1, TYDEX Co., Russia) and subsequently interacts with the first THz quarter-wave plate (QWP1, Product model: WP-CQ-D50-0W118.8-L/4, Operation wavelength range 40–1,000 μm, Material THz grade crystal quartz, TYDEX Co., Russia), collectively serving as the preselection components. These elements are aligned with their optical axes both at *π*/4 relative to the horizontal direction. By adjusting THz polarizer 1 relative to QWP1, small phase variations between the horizontal and vertical polarization components are induced. For postselection, we utilize the second THz quarter-wave plate (QWP2, Product model: WP-CQ-D50-0W118.8-L/4, Operation wavelength range 40–1,000 μm, Material THz grade crystal quartz, TYDEX Co., Russia) and the second THz polarizer 2 (Product model: PWO0S-012-050, THz wire grid polarizers, wavelength range above 150 μm, extinction ratio >1,000:1, TYDEX Co., Russia), both oriented at −*π*/4 relative to the horizontal direction. Rotation of THz polarizer 2 facilitates the construction of the postselection process described in eq. (4), yielding an imaginary weak value as detailed in eq. (5). The postselected THz wave is then detected using a THz detector to estimate phase variations.

In Figure 1b, the colored section signifies the quantum weak measurement segment along the entire optical path, which is the same optical path depicted in Figure 1a sans the two quarter-wave plates. Here, the THz wave first encounters the preselection component THz polarizer 1, with its optical axis set at an angle of *π*/4 relative to the horizontal direction. Adjusting THz polarizer 1 induces minute amplitude variations between horizontal and vertical polarization components. For postselection, we employ the second THz polarizer 2, aligned at −*π*/4 relative to the horizontal direction. By adjusting THz polarizer 2, the postselection process described in eq. (7) is established, resulting in a purely real weak value derived from eq. (8). The THz wave after postselection then undergoes measurement using a THz detector to quantify amplitude variations. All experiments were conducted under standard room temperature and humidity conditions to ensure that environmental factors did not influence the results.

**Figure 2:**
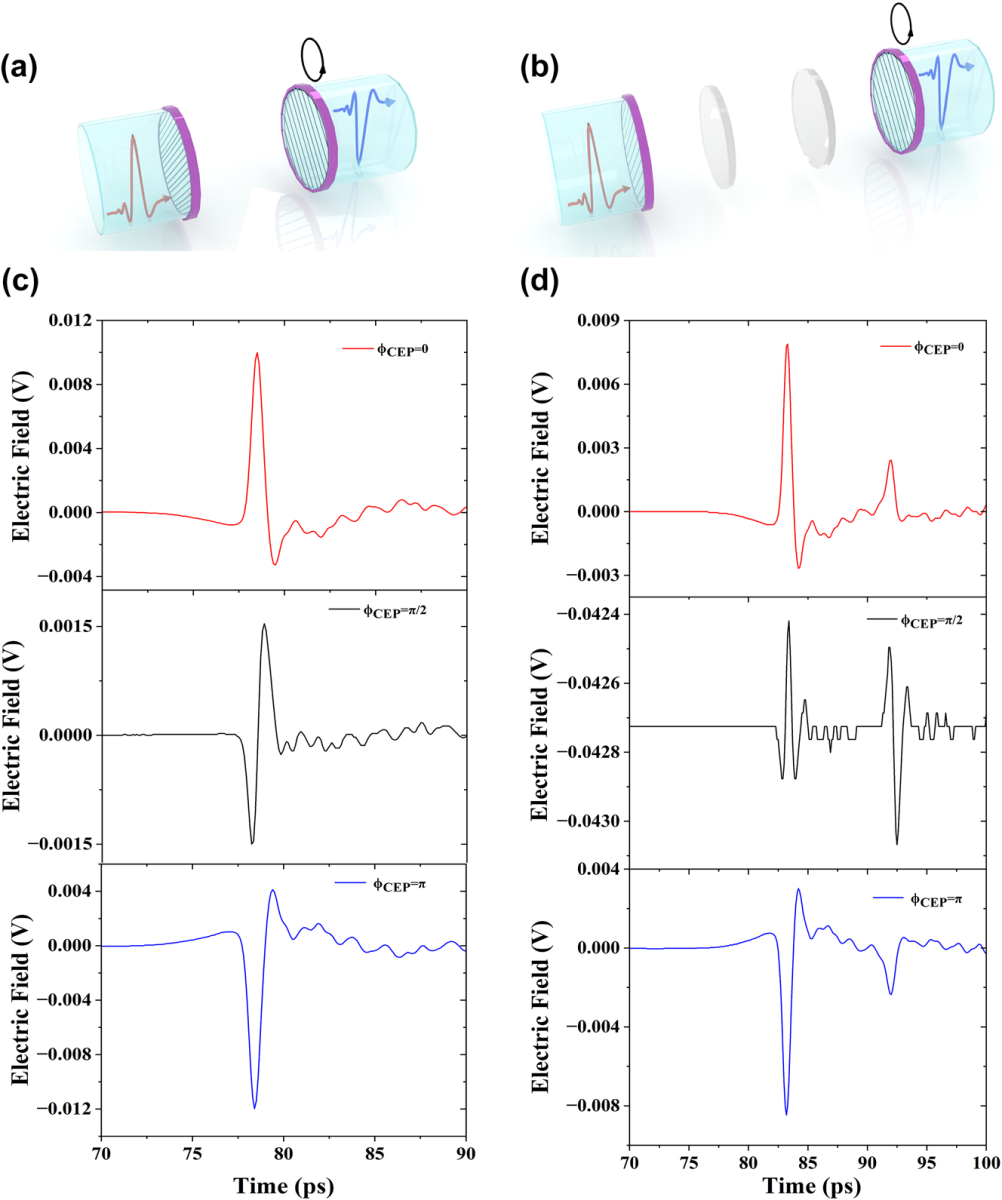
Different postselected states correspond to THz electric fields characterized by various CEPs of *ϕ*_CEP_ = 0, *π*/2, and *π*. (a) A schematic illustration of THz polarizer 1 and polarizer 2, depicting the rotation of THz polarizer 2 to prepare different postselected states. (b) A corresponding schematic for the THz electric fields associated with *ϕ*_CEP_ = 0, *π*/2, and *π* based on the configuration in part (a). (c) A schematic representation of THz polarizer 1, THz quarter-wave plates QWP1 and QWP2, along with THz polarizer 2, illustrating the rotation of THz polarizer 2 to generate different postselected states. (d) A corresponding schematic for the THz electric fields associated with *ϕ*_CEP_ = 0, *π*/2, and *π* as depicted in part (c).

In the terahertz system illustrated in Figure 1b, we employed various postselected states as depicted in Figure 2a. When the terahertz polarizer P2 is oriented orthogonally to THz polarizer P1, the phase *ϕ*_CEP_ in the initial state is *π*/2 (as shown in Figure 2c), where the positive and negative peak-to-peak values are nearly equal. Upon rotating the terahertz polarizer P2 by +4° around the orthogonal center, Figure 2c shows that the negative peak electric field exceeds the positive peak electric field, causing *ϕ*_CEP_ to shift to *π*. Conversely, when P2 is rotated −4° around the orthogonal center, the direction of the electric field is reversed, resulting in *ϕ*_CEP_ becoming 0, as illustrated in Figure 2c. Additionally, we utilized the terahertz system depicted in Figure 1a and set different postselected states as shown in Figure 2b. Notably, due to the presence of a quarter-wave plate, a reflection peak is observed at 92 ps. Furthermore, when THz polarizer P1 and P2 are orthogonal, *ϕ*_CEP_ is *π*/2 (as shown in Figure 2d), and the peak value of the reflected signal is greater than that of *ϕ*_CEP_ at both 0 and *π*, and it also surpasses the main peak at 83 ps. When the terahertz polarizer P2 is rotated ±4° around the orthogonal center, the behavior is consistent with that observed in Figure 2c. Thus, the CEP shift was effectively realized through the manipulation of terahertz polarizers P1 and P2, allowing for clear observation of the changes in the temporal waveforms of the THz pulse using this terahertz system. Figure S1 (Supplementary Materials) illustrates the terahertz time-domain electric field diagrams for the rotated QWP. Notably, compared to Figure 2, the change in electric field polarity is less pronounced when the polarizer is rotated by the same angle. The ability to manipulate terahertz waveforms is critical for a variety of applications, including terahertz scanning tunneling microscopy (THz-STM), which has traditionally employed pairs of spherical or cylindrical lenses or metamaterial arrays to achieve broadband THz CEP control [37], [38], [39].

**Figure 3:**
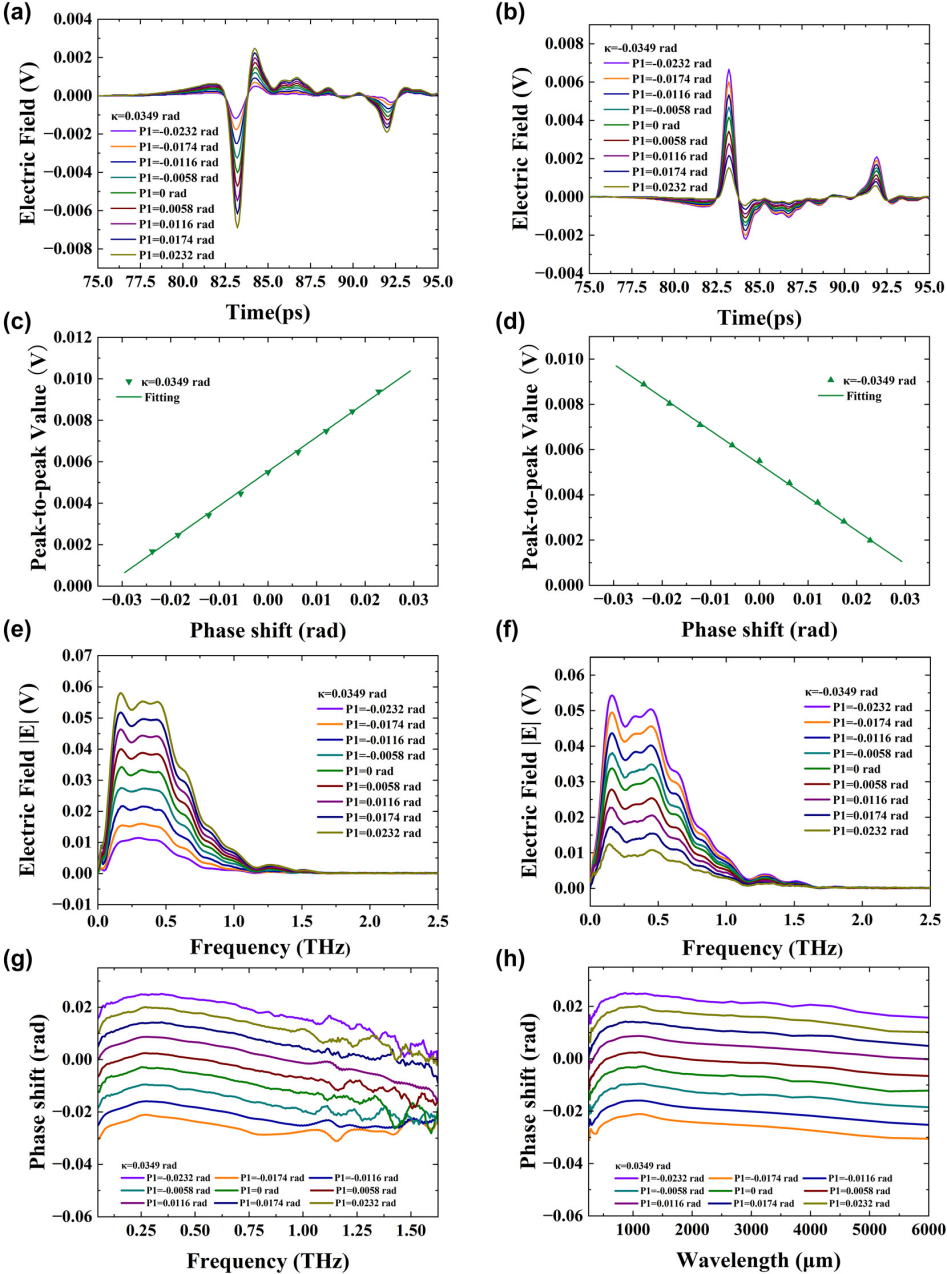
Experimental results in time and frequency domain show phase shift measurements with postselected angle *κ* = ±0.0349 rad. (a) Electric field with *κ* = 0.0349 rad in time domain. (b) Electric field with *κ* = −0.0349 rad in time domain. (c) Peak-to-peak signal with *κ* = 0.0349 rad in time domain. (d) Peak-to-peak signal with *κ* = −0.0349 rad in time domain. (e) Frequency domain spectroscopy with *κ* = 0.0349 rad. (f) Frequency domain spectroscopy with *κ* = −0.0349 rad. (g) Function of phase shift *α* and frequency. (h) Function of phase shift *α* and wavelength.

We first utilized the terahertz system depicted in Figure 1a to measure the phase shift. The postselected parameters were *κ* = 0.0349, 0.0698, and 0.0872 rad, selected three times. Each time, THz polarizer 1 was adjusted by 0.0058 rad for *κ* = 0.0349 rad, and by 0.0116 rad for *κ* = 0.0698 and 0.0872 rad. Figure 3a and b depicts the obtained time domain spectral change from 70 to 90 ps with an amplitude of *κ* = ±0.0349 rad, while Figure 3c and d shows the peak-to-peak value of the time domain signal. In Figure 3a and b, the 9 sampling curves of the time domain signal are spaced apart and do not overlap, even when THz polarizer 1 rotates by 0.0058 rad each time. Additionally, the THz quarter-wave plates QWP1 and QWP2 in the optical path cause multiple reflections due to incomplete parallelism, resulting in a reflection peak at 92 ps in the time domain signal. Furthermore, since the electric field is a vector, adjusting THz polarizer 2 by ±0.0349 rad after making THz polarizer 1 and THz polarizer 2 orthogonal causes the time domain signal to reverse. Figure 3c and d also show the peak-to-peak value changes linearly and in opposite directions with the continuous adjustment of THz polarizer 1. Figure 3c and d shows phase changes ranging from −0.0187 rad to 0.0183 rad, with intervals of 0.004 rad. In Figure 3a and b, the electric field magnitude is 10^−3^ V after applying pre- and postselection because the number of photons is greatly reduced. Before the pre- and postselection, the peak value of the electric field was approximately 0.35 V. The electric field strength $\left|E\right|$ of the terahertz detector is directly proportional to the formula intensity *I*, as expressed by the equation $\left|E\right|$ ∝ *I*. This relationship serves as the foundation for subsequent calculations. The sensitivity of the phase detection is calculated as $S_{\alpha }=\Delta \left(\left|E_{\alpha }^{+}\right|-\left|E_{\alpha }^{-}\right|\right)/\Delta \alpha =0.3133$ V/rad, and the standard deviation of the peak-to-peak value is measured as $\sigma \left|E_{\alpha }\right|=1.57\times 10^{-4}\;\text{V}$ , resulting in a terahertz wave phase resolution of $R_{\alpha }=\sigma \left|E_{\alpha }\right|/S_{\alpha }=5.02\times 10^{-4}\;\text{rad}$ . The phase change, sensitivity, and resolution of the terahertz quarter-wave plate are consistent starting at its 60 μm wavelength, eliminating the need for additional phase testing at single wavelengths. Figure 3e and f presents the frequency domain spectroscopy using time domain signals for fast Fourier transform (FFT). After the FFT, it can be seen that since electric field strength $\left|E\right|$ is a scalar, Figure 3e and f no longer have opposite directions except for the different order of signal size. By processing the frequency domain signal, Figure 3g and h displays the relationship between phase *α* and frequency with a range of 0.05–1.625 THz and wavelength with a range of 250–6,000 μm. In addition, Figure S2 (in the Supporting Information) shows the data for *κ* = ±0.0698 rad, and Figure S3 (in the Supporting Information) shows the data for *κ* = ±0.0872 rad, both of which are similar to Figure 3. These results indicate that the stability of our system ensures that the resolution of different postselected phases is within 10^−4^ rad. Our findings are consistent with previous studies using wide spectrum light (visible to infrared) for phase weak measurements, which also achieved a resolution of 10 ^−4^ rad [40], [41]. In contrast, Qiu et al. employed a monochromatic laser (He–Ne laser) and achieved phase delay estimation with a precision on the order of 10^−5^ rad [42]. This enhancement in precision can be attributed to the dispersion effects produced by broad spectrum light sources, which are significant factors limiting measurement accuracy in high-precision optical systems. Furthermore, the extinction ratio of our THz polarizer is greater than 1,000:1, whereas the Glan–Taylor polarizer employed in measurements using a monochromatic light source boasts an extinction ratio of 100,000:1. This disparity in extinction ratios further contributes to reduced measurement accuracy in our setup.

**Figure 4:**
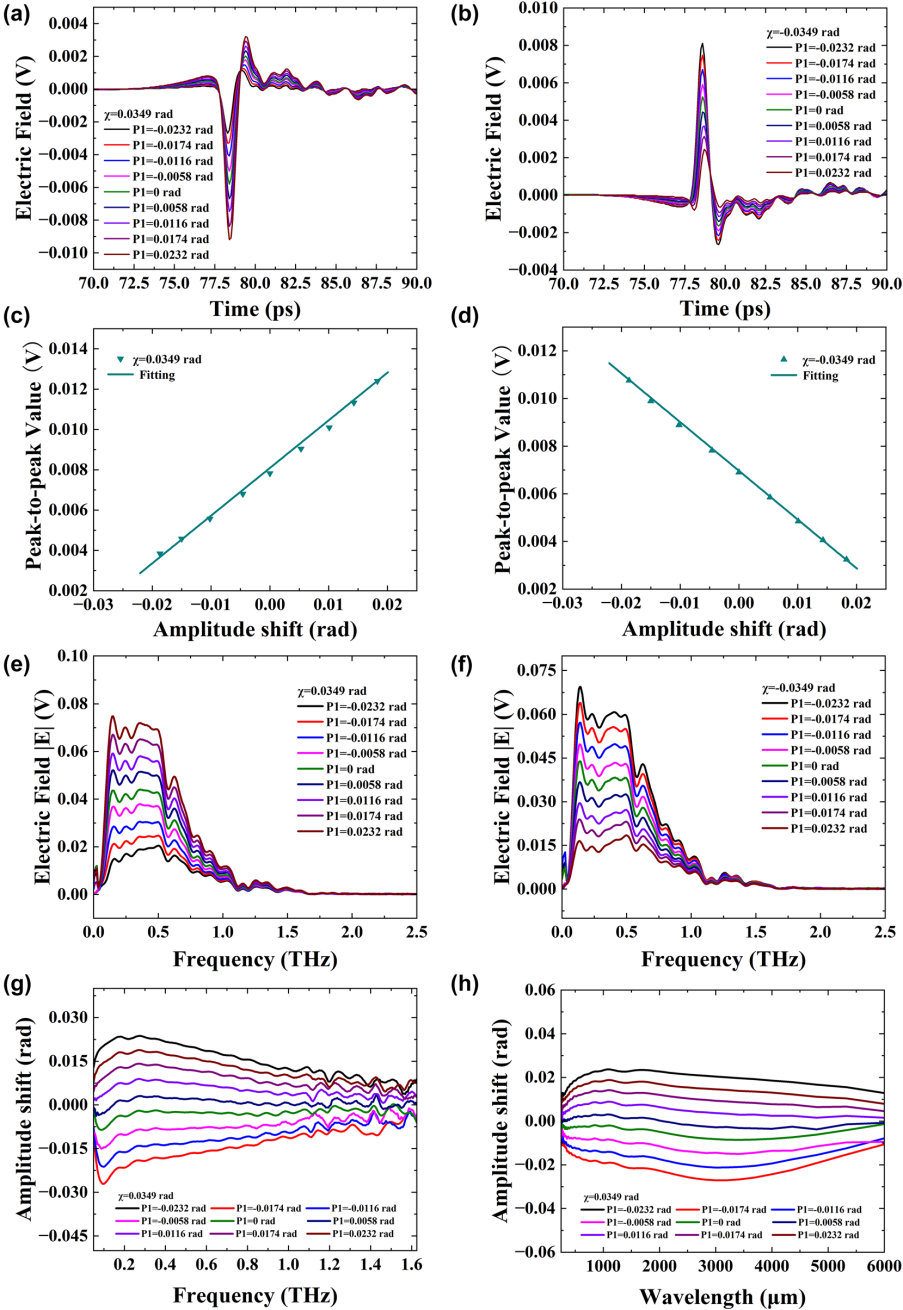
Experimental results in time and frequency domains show amplitude shift measurements with postselected angle *χ* = ±0.0349 rad. (a) Electric field with *χ* = 0.0349 rad in time domain. (b) Electric field with *χ* = −0.0349 rad in time domain. (c) Peak-to-peak signal with *χ* = 0.0349 rad in time domain. (d) Peak-to-peak signal with *χ* = −0.0349 rad in time domain. (e) Frequency domain spectroscopy with *χ* = 0.0349 rad. (f) Frequency domain spectroscopy with *χ* = −0.0349 rad. (g) Function of amplitude shift *β* and frequency. (h) Function of amplitude shift *β* and wavelength.

Next, we employed the terahertz system shown in Figure 1b to measure the amplitude shift. Interestingly, the experimental results were similar to those obtained using the setup in Figure 1a. The postselected parameters included *χ* = 0.0349, 0.0698, and 0.0872 rad, which were selected three different times. Each time, THz polarizer 1 was fine-tuned by 0.0058 rad when *χ* = 0.0349 rad, and by 0.0116 rad when *χ* = 0.0698 and 0.0872 rad. Figure 4a and b illustrates the change in the time domain with an amplitude of *χ* = ±0.0349 rad, and Figure 4c and d shows the peak-to-peak value of the time domain signal. In Figure 4a and b, the 9 sampling signal curves of the time domain signal are spaced apart and do not overlap, even when THz polarizer 1 rotates by 0.0058 rad each time. Additionally, the THz quarter-wave plates QWP1 and QWP2 are not in the optical path so that no multiple reflections due to incomplete parallelism results, thus no reflection peak appear at 92 ps in the time domain signal. Furthermore, same as in the previous analysis, since the electric field is a vector, adjusting THz polarizer 2 by ±0.0349 rad after making THz polarizer 1 and THz polarizer 2 orthogonal causes the time domain signal to reverse. Figure 4c and d also show that the peak-to-peak value changes linearly and in opposite directions with the continuous adjustment of THz polarizer 1. Figure 4c and d shows amplitude changes ranging from −0.0238 rad to 0.0228 rad, with intervals of 0.0056 rad. Using the formula, the sensitivity of the amplitude is calculated $S_{\beta }=\Delta \left(\left|E_{\beta }^{+}\right|-\left|E_{\beta }^{-}\right|\right)/\Delta \beta =0.4351\;\text{V}/\text{rad}$ , and the standard deviation of the peak-to-peak value is measured as $\sigma \left|E_{\beta }\right|=1.9\times 10^{-4}\;\text{V}$ , resulting in a terahertz wave amplitude resolution of $R_{\beta }=\sigma \left|E_{\beta }\right|/S_{\beta }=4.38\times 10^{-4}\;\text{rad}$ . The amplitude change, sensitivity, and resolution of the terahertz are consistent starting at its 60 μm wavelength, eliminating the need for additional amplitude testing at single wavelengths. Figure 4e and f presents the frequency domain spectroscopy using time domain signals for FFT. After FFT transformation, it can be seen that since electric field strength $\left|E\right|$ is a scalar, Figure 4e and f no longer have opposite directions except for the different order of signal size. By processing the frequency domain signal, Figure 4g and h displays the relationship between amplitude shift *β* and frequency with a range of 0.05–1.625 THz and wavelength with a range of 250–6,000 μm. In addition, Figure S4 (in the Supporting Information) shows the data for *χ* = ±0.0698 rad, and Figure S5 (in the Supporting Information) shows the data for *χ* = ±0.0872 rad, both of which are similar to Figure 4. These results also indicate that the stability of our system ensures that the resolution of different postselected amplitude is within 10^−4^ rad. It is important to note that the resolution of phase and amplitude measurements can be significantly affected by various sources of noise, primarily arising from the terahertz transmitter, delay line, terahertz detector, incident angle error of the terahertz wave, and fluctuations in the experimental environment. For instance, environmental factors such as temperature and humidity can impact the stability of terahertz signals, leading to additional noise. To minimize measurement errors, we have taken several steps in our experimental design. Specifically, the spectrum for each measurement is determined by averaging the results of three independent experiments. Each data collection consists of averaging 60,000 individual spectra collected over a 60-s timeframe. This approach helps to enhance the reliability and accuracy of our measurements by effectively reducing the impact of random noise from various sources.

**Figure 5:**
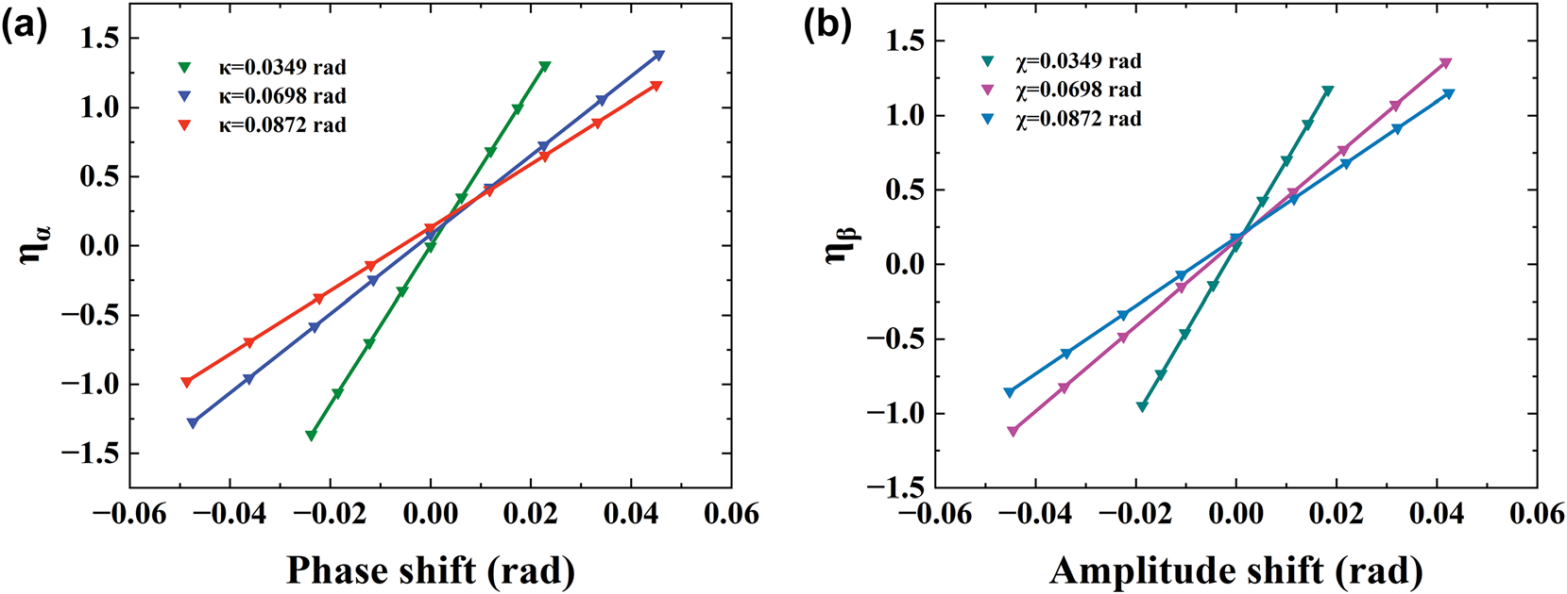
The contrast ratio *η*_*α, β*_ of different postselection parameters. (a) The contrast ratio *η*_*α*_ of the phase shift with different *κ* = 0.0349, 0.0698, and 0.0872 rad, respectively. (b) The contrast ratio *η*_*β*_ of the phase shift with different *χ* = 0.0349, 0.0698, and 0.0872 rad, respectively.

The postselection parameter *κ* ≪ 1 or *χ* ≪ 1 is fixed, although the THz wave electric field or intensity caused by phase or amplitude shift undergoes weak value amplification $\left(\left|A_{w\left(\alpha \right),\left(\beta \right)}^{\pm }\right|\right)$ , the overall attenuation of the THz wave electric field or intensity (sin^2^ *κ* or sin^2^ *χ*) leads to a general decrease in THz wave electric field or intensity ( $\text{si}{\text{n}}^{2}\kappa \left|A_{w\left(\beta \right)}^{\pm }\right|\approx \sin\;\kappa$ or $\text{si}{\text{n}}^{2}\chi \left|A_{w\left(\alpha \right)}^{\pm }\right|\approx \sin\;\chi$ ). So, how is the effect of linear weak value amplification manifested? To elucidate this point intuitively, we introduce the contrast ratio $\eta_{\alpha ,\beta }=\left(\left|E_{\alpha ,\beta }^{+}\right|-\left|E_{\alpha ,\beta }^{-}\right|\right)/\left(\left(\left|E_{\alpha ,\beta }^{+}\right|+\left|E_{\alpha ,\beta }^{-}\right|\right)/2\right)$ . From Figure 5a and b, we can see that as the postselection parameter decreases, the contrast ratio between phase and amplitude *η*_*α*,*β*_ increases. The contrast ratio *η*_*α*,*β*_ is proportional to the weak value $A_{w\left(\alpha \right),\left(\beta \right)}^{\pm }$ , where the case $\left|A_{w\left(\alpha \right),\left(\beta \right)}^{\pm }\right|=\cot\left(0.0349\right)$ represents the maximum slope for phase and amplitude shifts.

Based on the experimental data, we found that, within a certain range, a smaller postselection angle corresponds to a larger weak value. However, it is not the case that a smaller postselection angle will always result in greater magnification. For instance, Qiu et al. discovered that when postselection angles approach complete orthogonality to preselection [42], there is a maximum weak value, after which magnification decreases rapidly as the postselection angle continues to decrease.

Ding et al. previously developed dual-wavelength metasurfaces capable of independently controlling both phase and amplitude at specific terahertz wavelengths. However, this design suffers from significant wavelength dependence, which limits its effectiveness across a broader range of wavelengths. Additionally, the complexity of the design and manufacturing processes necessitates meticulous parameter adjustments and extensive simulations [43]. In contrast, Kakenov et al. utilized graphene in electrolyte-gated devices to modulate terahertz phase and amplitude. The performance of these devices is heavily influenced by the quality and consistency of the graphene used. Furthermore, electrolyte gating introduces potential stability issues, as these devices can be sensitive to environmental factors such as humidity and temperature. Achieving the necessary charge density for effective electrolyte gate control poses challenges due to the intricate nature of the manufacturing processes. Moreover, scaling production and integrating these technologies cost-effectively present significant hurdles, given the precision required in manufacturing [44]. In our experiment, we demonstrate a method applicable across a wide bandwidth, rather than being limited to a single wavelength, while effectively modulating both phase and amplitude. This approach avoids the need for complex structures, ensuring repeatability and stability. Notably, without additional control conditions, the modulation resolution for both phase and amplitude can reach 10^−4^ rad, which is significantly finer than the phase range (−*π* to *π*) and amplitude range (0–1) achieved in the previously discussed methods.

To verify the theory and the polarization-sensitive phase and amplitude THz measurement system, we used chiral samples in the next phase of our experiments. Chirality, the property of molecular asymmetry where molecules cannot be superimposed onto their mirror images, is prevalent in nature [45], [46]. Chiral molecules exhibit different biochemical behaviors from their mirror images, making them crucial in various biological processes. When a linearly polarized light beam passes through a chiral sample, it exhibits optical activity (OA), which comprises ORD and CD. OA refers to the interaction of chiral enantiomers with polarized light, evident in CD and ORD. CD measures the differential absorption of left (LCP) and right (RCP) circularly polarized light, corresponding to phase variation. ORD, on the other hand, measures the differential refraction, corresponding to amplitude variation. The spectra of both CD and ORD differentiate enantiomers by their sign and can be linked to absolute molecular configurations. Such interactions can be exploited as powerful tools for detecting chiral molecules, known as chiral sensing. Optical chiral sensing is advantageous because it is easy to implement and noninvasive to analytes. However, it presents challenges due to the extremely weak chiroptical signals of natural molecules (10^−4^ – 10^−6^ that of optical absorption), and that such weak signals coexist with much stronger signals of achiral absorptions.

CD and ORD correspond to the imaginary and real parts of the complex chiral susceptibility, much like how ordinary absorption and dispersion correspond to the imaginary and real parts of the complex refractive index [8]. To characterize both the imaginary (CD) and real (ORD) parts of the chiroptical susceptibility simultaneously, we will use our THZ-VWA system to construct an imaginary weak value and a real weak value.

**Figure 6:**
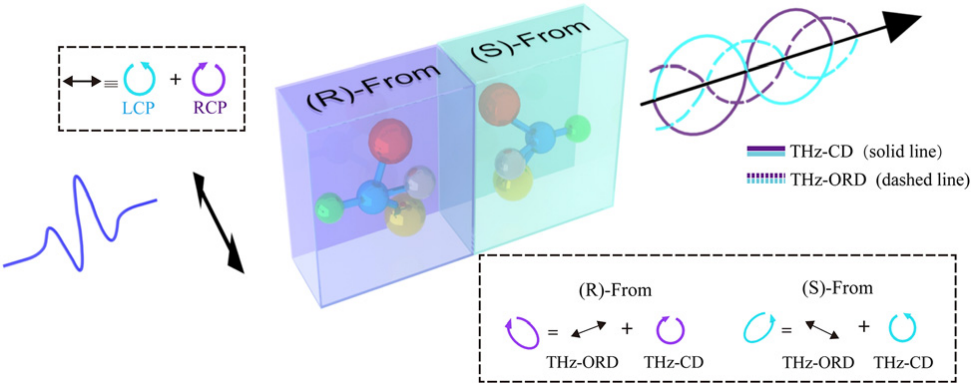
Simplified diagram of the basic principle of THz-ORD and THz-CD measurements includes the process of changing the polarization state. The THz wave is linearly polarized before interacting with a chiral sample. THz-ORD describes the difference in the propagation speeds of left- and right-circularly polarized light, whereas THz-CD refers to the difference in light absorption between these two polarizations.

Figure 6 depicts the scenario when linearly polarized terahertz light (an equal superposition of left circularly polarized (LCP) and right circularly polarized (RCP) light) passes through a sample with opposite chiral properties [6]. The THz-ORD and THz-CD signals arise due to differences in the refractive index and absorption coefficient. After the light interacts with the two different enantiomers, the (R)-form (purple) and the (S)-form (blue), these perpendicular components are out of phase with each other. If the change is small, the individual THz-CD (solid line) and THz-ORD (dashed line) components are approximately orthogonal.

The polarization state of THz wave is preselected as follows

(10) $$\begin{array}{c} \left|\psi_{\text{pre}}\right\rangle =\frac{1}{\sqrt{2}}\left(\left|H\right\rangle +\left|V\right\rangle \right)=\frac{1}{2}\left(\left(1-i\right)\left|L\right\rangle +\left(1+i\right)\left|R\right\rangle \right), \end{array}$$

where

(11) $$\begin{array}{c} \left|L\right\rangle =\frac{1}{\sqrt{2}}\left(\left|H\right\rangle +i\left|V\right\rangle \right), \end{array}$$

(12) $$\begin{array}{c} \left|R\right\rangle =\frac{1}{\sqrt{2}}\left(\left|H\right\rangle -i\left|V\right\rangle \right), \end{array}$$

where, we decompose the *π*/4 linearly polarized light into left-handed and right-handed circularly polarized light with equal amplitudes. $\left|L\right\rangle$ and $\left|R\right\rangle$ represent the states of left-handed circularly polarized light and right-handed circularly polarized light, respectively. And as before, $\left|H\right\rangle$ and $\left|V\right\rangle$ denote the horizontal and vertical polarization states, respectively.

When a linearly polarized THz wave passes through chiral molecules, differences in refractive index and absorption coefficient introduce small phase and amplitude changes between the left-handed and right-handed circularly polarized components. These changes are known as ORD and CD signals, respectively. ORD causes an amplitude shift in the beam, while CD causes a phase change. Therefore, the operators for measuring amplitude and phase can be used to describe the operators for ORD and CD.

(13) $$\begin{array}{c} \hat{U}=\exp\left(\frac{\theta \left(\lambda \right)+i\delta \left(\lambda \right)}{2}\hat{A}\right), \end{array}$$

where, the ORD and CD signals of the sample are represented by the following equations:

(14) $$\begin{array}{c} \theta \left(\lambda \right)=\frac{2\pi l\left(n_{L}-n_{R}\right)}{\lambda }, \end{array}$$

(15) $$\begin{array}{c} \delta \left(\lambda \right)=\frac{\ln\left(10\right)\left(\varepsilon_{L}-\varepsilon_{R}\right)Cl}{2}, \end{array}$$

in these equations, $\varepsilon_{L\left(R\right)}$ represents the molar extinction coefficient, $n_{L\left(R\right)}$ represents the refractive index, *λ* represents the wavelength, *C* represents the concentration, and *l* represents the optical path length. Also, $\hat{A}=\left|L\right\rangle \left\langle L\right|-\left|R\right\rangle \left\langle R\right|$ is the observable quantity of the two-level quantum system.

After the beam passes through the chiral sample, the state of the system changes to

$$\begin{array}{ll} \left|\psi^{\prime }\right\rangle & =\hat{U}\left|\psi_{\text{pre}}\right\rangle \\ & =\exp\left(\frac{\theta \left(\lambda \right)+i\delta \left(\lambda \right)}{2}\hat{A}\right)\frac{1}{2}\left(\left(1-i\right)\left|L\right\rangle +\left(1+i\right)\left|R\right\rangle \right) \end{array}$$

(16) $$\begin{array}{ll} & \;=\frac{1}{2}\left(\exp\left(\frac{\theta \left(\lambda \right)+i\delta \left(\lambda \right)}{2}\right)\left(1-i\right)\left|L\right\rangle \right.\\ & \;\left.+\exp\left(-\frac{\theta \left(\lambda \right)+i\delta \left(\lambda \right)}{2}\right)\left(1+i\right)\left|R\right\rangle \right), \end{array}$$

in the weak measurement model, $\theta \left(\lambda \right)\ll 1$ and $\delta \left(\lambda \right)\ll 1$ .

Subsequently, the ORD signal is measured by constructing a postselected polarization state with a real weak value, similar to the amplitude measurement above

(17) $$\begin{array}{c} \left|\psi_{\text{post}\left(\theta \left(\lambda \right)\right)}^{\pm }\right\rangle =\cos\left(\frac{\pi }{4}\mp \chi \right)\left|L\right\rangle -\sin\left(\frac{\pi }{4}\mp \chi \right)\left|R\right\rangle . \end{array}$$

According to the general expression for the weak value, the real weak value is derived from the preselected and postselected states

(18) $$\begin{array}{c} A_{w\left(\theta \left(\lambda \right)\right)}^{\pm }=\frac{\left\langle \psi_{\text{post}\left(\theta \left(\lambda \right)\right)}^{\pm }\left|\hat{A}\right|\psi_{\text{pre}}\right\rangle }{\left\langle \psi_{f\left(\theta \left(\lambda \right)\right)}^{\pm }\left|\right.\;\psi_{\text{pre}}\right\rangle }=\pm \cot\chi . \end{array}$$

The intensity of the postselected THz wave corresponding to the state is given by

$$I_{\theta \left(\lambda \right)}^{\pm }=I_{0}{\left|\left\langle \psi_{\text{post}\left(\theta \left(\lambda \right)\right)}^{\pm }\left|\exp\left(\frac{\theta \left(\lambda \right)+i\delta \left(\lambda \right)}{2}\hat{A}\right)\right|\psi_{\text{pre}}\right\rangle \right|}^{2}$$

$$\;\approx I_{0}{\left|\left\langle \psi_{\text{post}\left(\theta \left(\lambda \right)\right)}^{\pm }\left|\right.\psi_{\text{pre}}\right\rangle \right|}^{2}\left[1+\theta \left(\lambda \right)\text{Re}\left(A_{w\left(\theta \left(\lambda \right)\right)}^{\pm }\right)\right]$$

(19) $$\begin{array}{c} =I_{0}\text{si}{\text{n}}^{2}\chi \left[1+\theta \left(\lambda \right)\text{Re}\left(A_{w\left(\theta \left(\lambda \right)\right)}^{\pm }\right)\right],\; \end{array}$$

Here, *I*_0_ denotes the initial THz intensity before postselection. In eq. (19), the approximation is based on the condition that ${\left|A_{w\left(\theta \left(\lambda \right)\right)}^{\pm }\right|}^{2}\left(\delta {\left(\lambda \right)}^{2}+\theta {\left(\lambda \right)}^{2}\right)/4\ll 1$ and *χ* ≪ 1.

Then, the CD signal is measured by constructing a postselected polarization state with an imaginary weak value

(20) $$\begin{array}{c} \left|\psi_{\text{post}\left(\delta \left(\lambda \right)\right)}^{\pm }\right\rangle =\frac{1}{\sqrt{2}}\left[\exp\left(\mp i\kappa \right)\left|L\right\rangle -\exp\left(\pm i\kappa \right)\left|R\right\rangle \right], \end{array}$$

± represents two symmetric postselection states. According to the weak value calculation formula, the imaginary weak value is derived from the preselected and postselected states

(21) $$\begin{array}{c} A_{w\left(\delta \left(\lambda \right)\right)}^{\pm }=\frac{\left\langle \psi_{\text{post}\left(\delta \left(\lambda \right)\right)}^{\pm }\left|\hat{A}\right|\psi_{\text{pre}}\right\rangle }{\left\langle \psi_{\text{post}\left(\delta \left(\lambda \right)\right)}^{\pm }\left|\right.\psi_{\text{pre}}\right\rangle }=\mp i\cot\kappa . \end{array}$$

The intensity of the postselected THz wave corresponding to the state is given by

$$I_{\delta \left(\lambda \right)}^{\pm }=I_{0}{\left|\left\langle \psi_{\text{post}\left(\delta \left(\lambda \right)\right)}^{\pm }\left|\exp\left(\frac{\theta \left(\lambda \right)+i\delta \left(\lambda \right)}{2}\hat{A}\right)\right|\psi_{\text{pre}}\right\rangle \right|}^{2}$$

$$\;\approx I_{0}{\left|\left\langle \psi_{\text{post}\left(\delta \left(\lambda \right)\right)}^{\pm }\left|\right.\psi_{\text{pre}}\right\rangle \right|}^{2}\left[1-\delta \left(\lambda \right)\text{Im}\left(A_{w\left(\delta \left(\lambda \right)\right)}^{\pm }\right)\right]$$

(22) $$\begin{array}{c} =I_{0}\text{si}{\text{n}}^{2}\kappa \left[1-\delta \left(\lambda \right)\text{Im}\left(A_{w\left(\delta \left(\lambda \right)\right)}^{\pm }\right)\right],\; \end{array}$$

Here, *I*_0_ denotes the initial THz intensity before postselection. In eq. (22), the approximation is based on the condition that ${\left|A_{w\left(\delta \left(\lambda \right)\right)}^{\pm }\right|}^{2}\left(\delta {\left(\lambda \right)}^{2}+\theta {\left(\lambda \right)}^{2}\right)/4\ll 1$ , *κ* ≪ 1.

**Figure 7:**
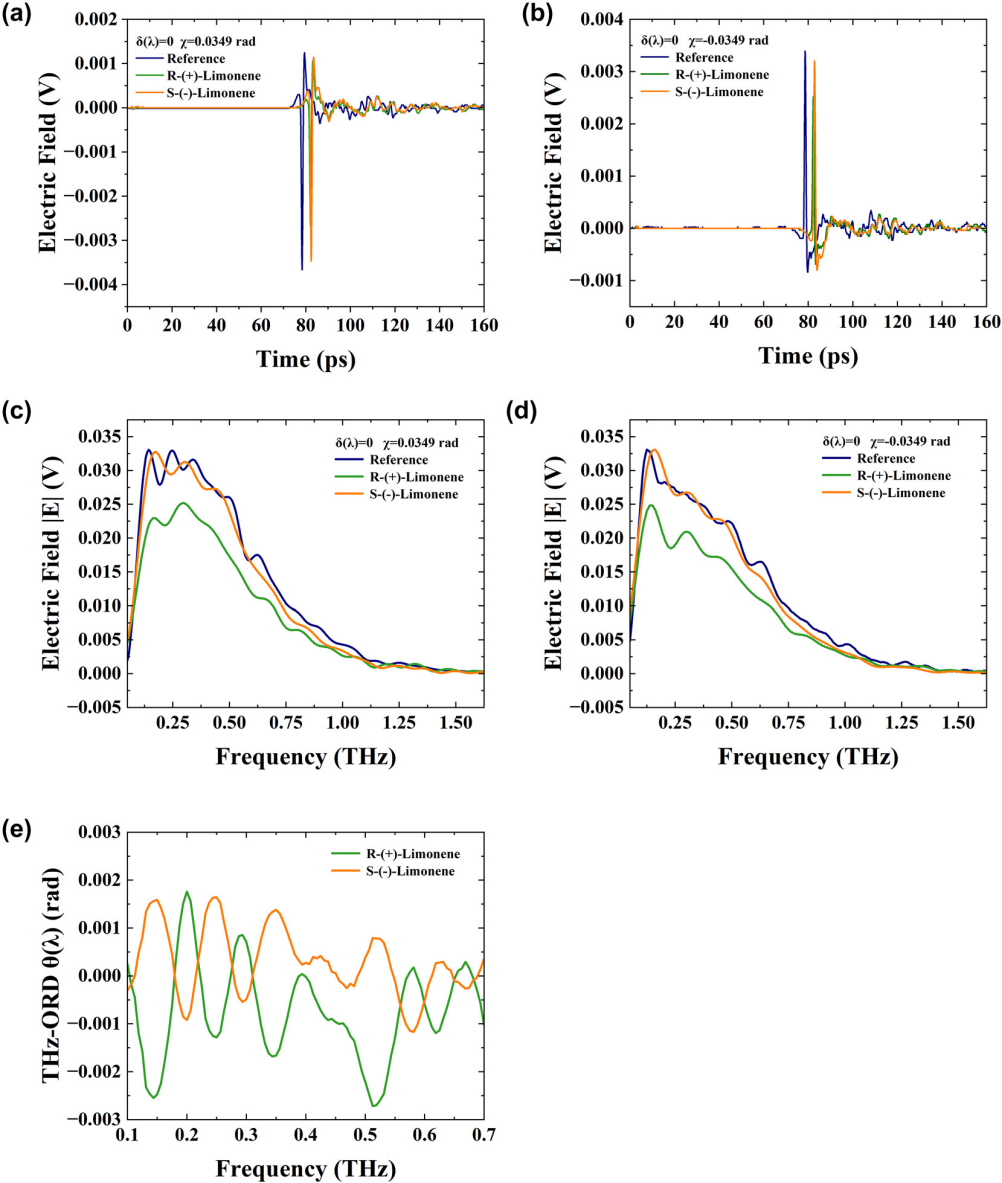
Ultra-broadband THz wave measurement of (R)- and (S)-limonene ORD spectra with postselected angles *χ* = ±0.0349 rad. (a) The time-domain electric field corresponding to *χ* = 0.0349 rad. (b) The time-domain electric field corresponding to *χ* = −0.0349 rad. (c) Frequency domain spectroscopy for *χ* = 0.0349 rad. (d) Frequency domain spectroscopy for *χ* = −0.0349 rad. (e) (R)-limonene absorption spectra. (f) (S)-limonene absorption spectra. (g) (R)- and (S)-limonene THz-ORD spectra.

We first utilized the THz-WVA system to measure the THz-ORD spectra of (R)- and (S)-limonene at *δ*(*λ*) = 0, with postselected parameters *χ* = 0.0349 rad (in Figure 5, the postselected parameter *χ* = 0.0349 rad is the largest weak value). The (R)- and (S)-limonene (C_10_H_16_) employed in the experiment are both PYRAM brand products, each with a purity exceeding 95 % and a molecular weight of 136.23 g/mol. Both limonenes are in liquid form and were sourced from Shanghai Bohu Biotechnology Co., Ltd. The sample cell’s window material is TPX, sourced from Dongguan Daehan Polymer Plastic Co., Ltd, China, with a diameter of 40 mm and a thickness of 2 mm. The gasket, with an inner diameter of 35 mm, an outer diameter of 39 mm, and a thickness of 2.5 mm, provides an optical path length of 2.5 mm for the (R)- and (S)-limonene samples. Figure 7a and b shows time domain changes from 0 to 160 ps with an amplitude of *χ* = ±0.0349 rad, while Figure 7c and d shows the frequency domain signal after performing FFT on the time domain signal. Figure 7a and b reveals that after adding 2.3 mL of (R)- and (S)-limonene, there is a time delay compared to the empty sample cell. Since (R)- and (S)-limonene are equal in volume, their signals overlap in the time domain. As before, since the electric field is a vector, adjusting THz polarizer 2 by ±0.0349 rad after making THz polarizer 1 and THz polarizer 2 orthogonal causes the time domain signal to reverse. Additionally, the time domain peak-to-peak values of (R)- and (S)-limonene differ due to their different terahertz absorption coefficients and refractive indices for left and right circularly polarized light. The change in optical rotation electric field $\left|E\right|$ for (R)-limonene is 7.05 × 10^−5^ V and for (S)-limonene is −3.25 × 10^−5^ V, indicating that the optical rotation directions of (R)- and (S)-limonene are opposite. Clearly, the change in electric field $\left|E\right|$ due to absorption is much greater than that due to optical rotation. This discrepancy explains why traditional terahertz systems cannot directly measure chiral samples. However, weak value amplification can amplify weak signals by several orders of magnitude while simultaneously suppressing technical noise to an extremely low level. This enhancement improves measurement resolution, enabling the effective measurement of chiral samples. Figure 7c and d presents the frequency domain spectra after FFT of the time domain signals. Processing the frequency domain signal, Figure 7e displays the THz-ORD spectra of (R)- and (S)-limonene, showing the relationship between *θ*(*λ*) and frequency from 0.1 to 0.7 THz, with (R)- and (S)-limonene ORD components being approximately orthogonal.

**Figure 8:**
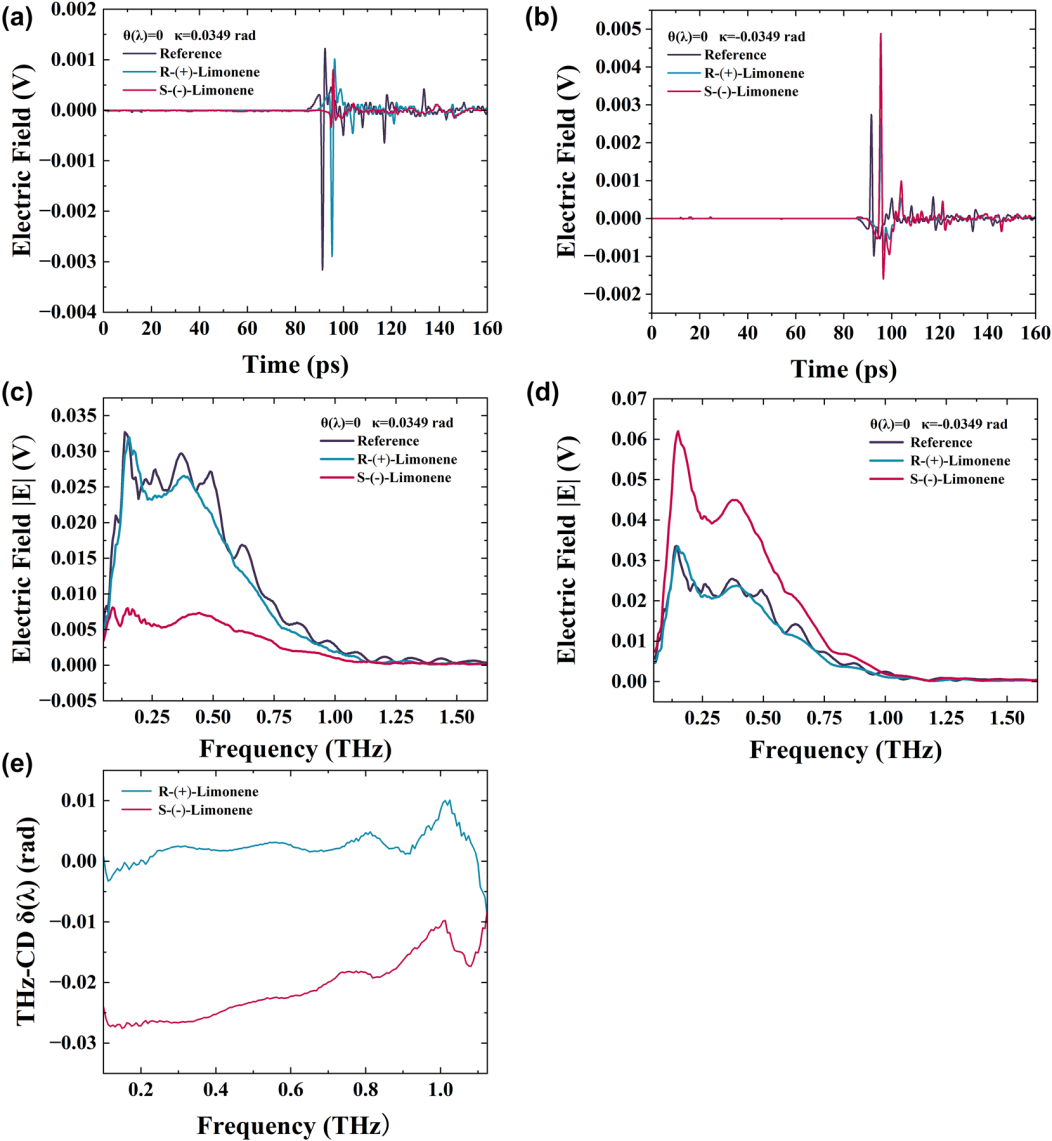
Ultra-broadband THz wave measurement of (R)- and (S)-limonene CD spectra with postselected angles *κ* = ±0.0349 rad. (a) The time-domain electric field corresponding to *κ* = 0.0349 rad. (b) The time-domain electric field corresponding to *κ* = −0.0349 rad. (c) Frequency domain spectroscopy for *κ* = 0.0349 rad. (d) Frequency domain spectroscopy for *κ* = −0.0349 rad. (e) (R)- and (S)-limonene THz-CD spectra.

We then utilized the THZ-WVA system to measure the THz-CD spectra of (R)- and (S)-limonene at *θ*(*λ*) = 0, with postselected parameters *κ* = 0.0349 rad. Figure 8a and b shows time domain changes from 0 to 160 ps with an amplitude of *κ* = ±0.0349 rad, while Figure 8c and d displays the frequency domain signal after performing FFT on the time domain signal. Figure 8a and b reveals that after adding 2.3 mL of (R)- and (S)-limonene, there is a time delay compared to the empty sample cell. Since (R)- and (S)-limonene are equal in volume, their signals overlap in the time domain. Additionally, the time domain peak-to-peak values of (R)- and (S)-limonene differ due to their different terahertz absorption coefficients and absorption of left and right circularly polarized light. Processing the frequency domain signal, Figure 8e displays the THz-CD spectra of (R)- and (S)-limonene, showing the relationship between *δ*(*λ*) and frequency from 0.1 to 1.125 THz, with the THz-CD components of (R)- and (S)-limonene being approximately orthogonal. It can be seen that the THz-CD spectrum of (R)-limonene has some data in the order of magnitude of 10^−5^ rad, which further explains why traditional terahertz methods cannot directly measure the THz-CD signal.

Since CCl_4_ as solvent for limonene is toxic that our laboratory is not ventilated to handle, we did not conduct tests on different concentrations of (R)- and (S)-limonene as of this writing.

**Figure 9:**
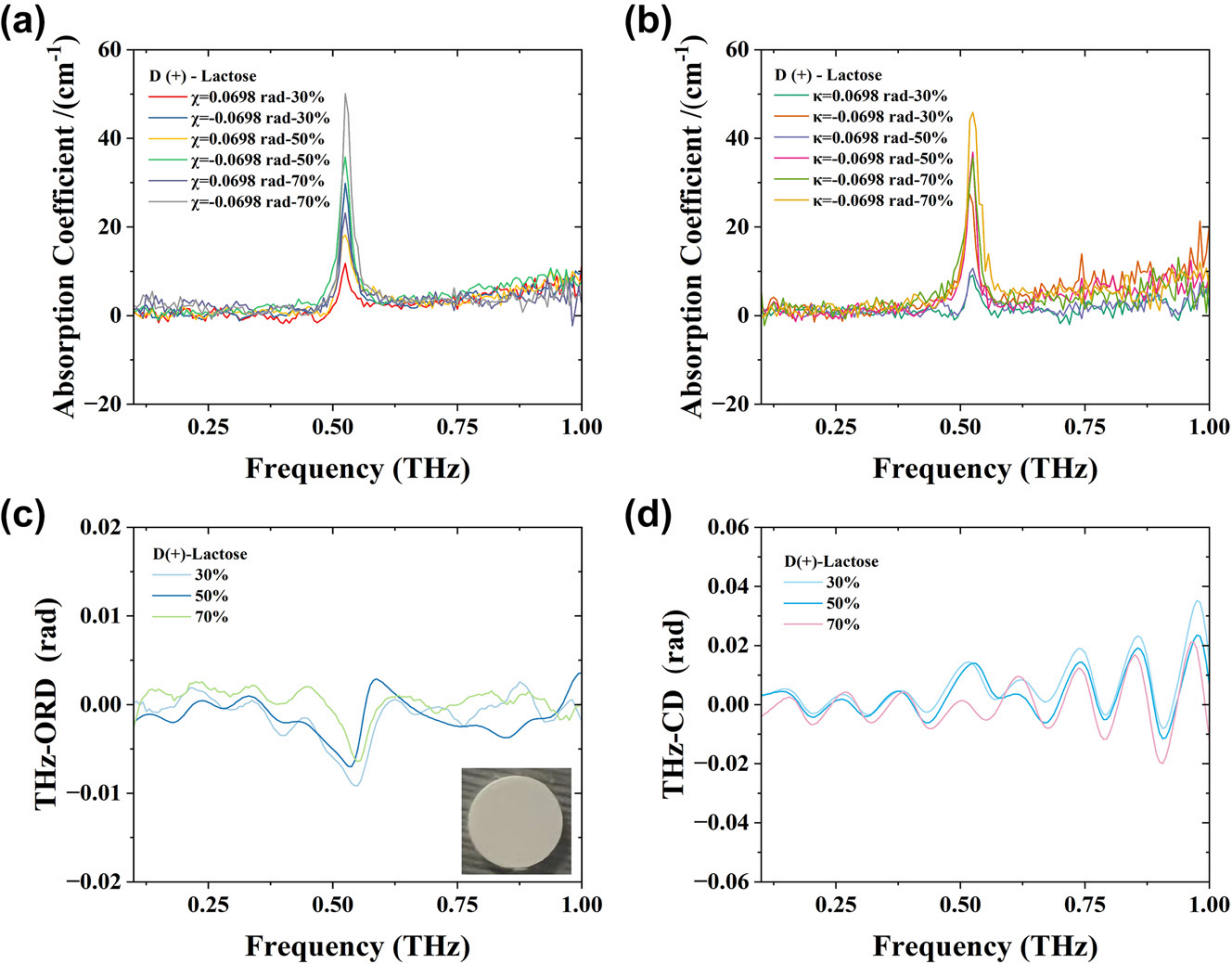
THz spectroscopic analysis of D(+)-lactose tablets. (a) D(+)-lactose absorption coefficient of 30 %, 50 %, and 70 % when *χ* = ±0.0698 rad; inset shows an optical image of a sample tablet. (b) D(+)-lactose absorption coefficient of 30 %, 50 %, and 70 % when *κ* = ±0.0698 rad. (c) D(+)-lactose THz-ORD spectra. (d) D(+)-lactose THz-CD spectra.

We also utilized this THz system to test D(+)-lactose (C_12_H_22_O_11_), which was sourced from Shanghai Yuanye Biotechnology Co., Ltd., with a purity exceeding 98 %. No further purification was performed prior to its use. D(+)-lactose and polyethylene were thoroughly ground and mixed in weight ratios of 30 %, 50 %, and 70 %, resulting in a total mass of 150 mg. A slicer was employed to apply a pressure of 12 tons/cm^2^ for 2 min, yielding slices with a diameter of 13 mm and a thickness ranging from 1 to 1.3 mm. Measurements were conducted using the optical path depicted in Figure 1. Figure 9a and b illustrates the absorption coefficient of lactose. Notably, under the same postselection conditions, the absorption coefficient increases with mass fraction, with the 70 % lactose sample exhibiting the highest absorption coefficient. In the absence of weak measurement techniques, the peak of the original time-domain signal exceeded that of the lower portion, with the absorption peak for 70 % lactose recorded at 0.53 THz and 20 cm^−1^. Upon reversing the electric field in the time domain when *ϕ*_CEP_ = 0 (as seen in Figure 2c), the absorption coefficient of 70 % lactose reached 50 cm^−1^ at 0.53 THz, reflecting a 150 % increase compared to the original value, as shown in Figure 9a. Conversely, when *ϕ*_CEP_ = 0 (Figure 2d), the absorption coefficient of 70 % lactose reached 45 cm^−1^ at 0.53 THz, indicating a 125 % increase compared to the original value, as shown in Figure 9b. The most significant absorption coefficient for 70 % lactose was observed when *χ* = −0.0698 rad and *κ* = −0.0698 rad, while enhancements were less pronounced at *χ* = 0.0698 rad or *κ* = 0.0698 rad.

The absorption coefficient of lactose is significantly influenced by the direction of the terahertz electric field, which can be attributed to several interrelated factors. First, the direction of the electric field alters the polarization degree of the lactose molecules, thereby affecting their response to the field. Second, the direction of the electric field determines the orientation of the molecules in space, and this orientation can influence the interactions between molecules. When the direction of the electric field changes, the resonance conditions of lactose may also shift, potentially leading to changes in the position and intensity of the absorption peaks. Additionally, the direction of the electric field affects the electronic charge distribution within the molecules, further altering their absorption characteristics. In summary, these factors interact with one another, resulting in a significant impact of the direction of the terahertz electric field on the absorption coefficient of lactose. Additionally, the absorption peak obtained without the addition of a quarter-wave plate in the postselection process (as illustrated in Figure 9a) was higher, characterized by a smooth waveform and a prominent peak. These observations suggest that the absorption peak of lactose is influenced not only by its mass, moisture content, and temperature [47] but also by the orientation of the terahertz electric field. Figure 9c and d presents the spectrum and circular dichroism analysis of lactose, respectively. In future experiments, we plan to include an additional set of lenses to focus the THz beam onto the sample tablet for testing and comparison.

To aid the evaluation of sensor performance in this study, we compared it with representative studies on chiral sensing as summarized in Table 1. Our approach involves an optical path with two polarizers for measuring chiral liquids, contrasting with methods utilizing metal metamaterial structures, which we did not employ. It is noteworthy that large chiral nanostructures may potentially obscure molecular chiral signals, thus limiting their practical application prospect [48]. Additionally, metamaterials, once designed, are not easily modifiable, and their preparation is complex. Despite their ability to amplify signals such as CD and ORD by several orders of magnitude, they also introduce noise. Even after removing background signals, the obtained chiral sample signal often deviates significantly from the true value. This is because chiral light effects (CD and ORD) are very weak, typically only 10^−6^ to 10^−4^ of the nonchiral background absorption signal.

**Table 1:** Performance parameter comparison for recently reported chiral sensing.

Sensor type	Characterization parameter and signal range	Analyte	Chiral sample state	Operation mode	Ref.
Pancharatnam–Berry metasurface	Circular dichroism (−11.6° to 16.4°)	Tyrosine	Tablet	Transmission	[49]
Chiral metamaterials	Circular dichroism (−40° to 40°)	Cyclotyrosine	Biocrystals	Transmission	[50]
Metal metasurface	Circular dichroism (−45° to 45°) and optical rotatory dispersion (90° to 90°)	Amino acids	Liquid	Reflection	[5]
THz-WVA	Circular dichroism (−0.02 to 0.04 rad) and optical rotatory dispersion (−0.01 to 0.003 rad)	Limonene, lactose	Liquid, tablet	Transmission	This work

## 4 Conclusions

In summary, we have presented a novel setup for THz broadband measurements of phase and amplitude variations in both the time and frequency domains using weak value amplification. Through tailored postselection parameters, we employed real weak values to precisely measure amplitude shifts. Furthermore, by integrating a THz quarter-wave plate into the setup, we effectively enlarged the postselection parameters space and were able to utilize imaginary weak values to accurately measure phase shifts. This enables us to deduce the complete polarization state variation of THz waves using the novel WVA scheme. Our method achieves a resolution of approximately 10^−4^ rad for both phase and amplitude variations in the time domain using the peak-to-peak value and determines phase and amplitude variations with frequency and wavelength in the frequency domain through the electric field $\left|E\right|$ spectrum. Furthermore, we successfully detected small signals of large-volume (R)- and (S)-limonene and D(+)-lactose through CD and ORD, overcoming the significant limitation of traditional THz system, which cannot directly measure the chiral optical activity of liquids and tablets. At the same time, effective manipulation of CEP offset was achieved in terahertz polarizers P1 and P2. When *ϕ*_CEP_ = 0, using weak measurement techniques can greatly enhance the absorption coefficient of lactose. It was found that the absorption coefficient of lactose is influenced not only by concentration, temperature, and water content but also by the orientation of the terahertz electric field. Given its general applicability, our approach to measuring broadband THz polarization phase and amplitude and optical activity offers simplicity, affordability, and robustness, enabling a wide range of experiments beyond research laboratories.

## Supplementary Material

Supplementary Material Details
