# Supplementary material for: Quantum weak value amplified terahertz chiroptical measurement
Source: Nanophotonics. 2025 Apr 16;14(12):2133–49. doi: 10.1515/nanoph-2024-0685 (PMC12147555; doi:10.1515/nanoph-2024-0685)
Supplement: Supplementary file 1 — Supplementary Material Details [file j_nanoph-2024-0685_suppl_001.docx]

**Supporting information**

**Quantum Weak Value Amplified Terahertz Chiroptical Measurement**

Liping Xu, ^1^ Jiangtao Xu, ^1^ Xin Yao, ^1^ Rumin Zhang,^1^ Gang Wen, ^1^ Lei Wang, ^1^ Xingxing Lu, ^2^ Zaoxia Li, ^2^ Wenquan Liu,^2^ Dongshan Wei, ^2^ Xiaoli Li, ^1^ Tianying Chang, ^2*^ and Hong-Liang Cui^2^

*Corresponding authors: [ty.chang@siat.ac.cn](mailto:ty.chang@siat.ac.cn)

**1. Terahertz time-domain electric field diagrams for rotated quarter-wave plate corresponding to carrier-envelope phases**

The following figure illustrates the terahertz time-domain electric field diagrams corresponding to the carrier-envelope phases (CEPs) for a rotated quarter-wave plate. Figure S1a shows a rotation of -0.0698 rad relative to the initial position, while Figure S1b presents the terahertz time-domain electric field diagram at the initial position without rotation. Figure S1c depicts the terahertz time-domain electric field diagram after a 0.0698 rad rotation relative to the initial position.


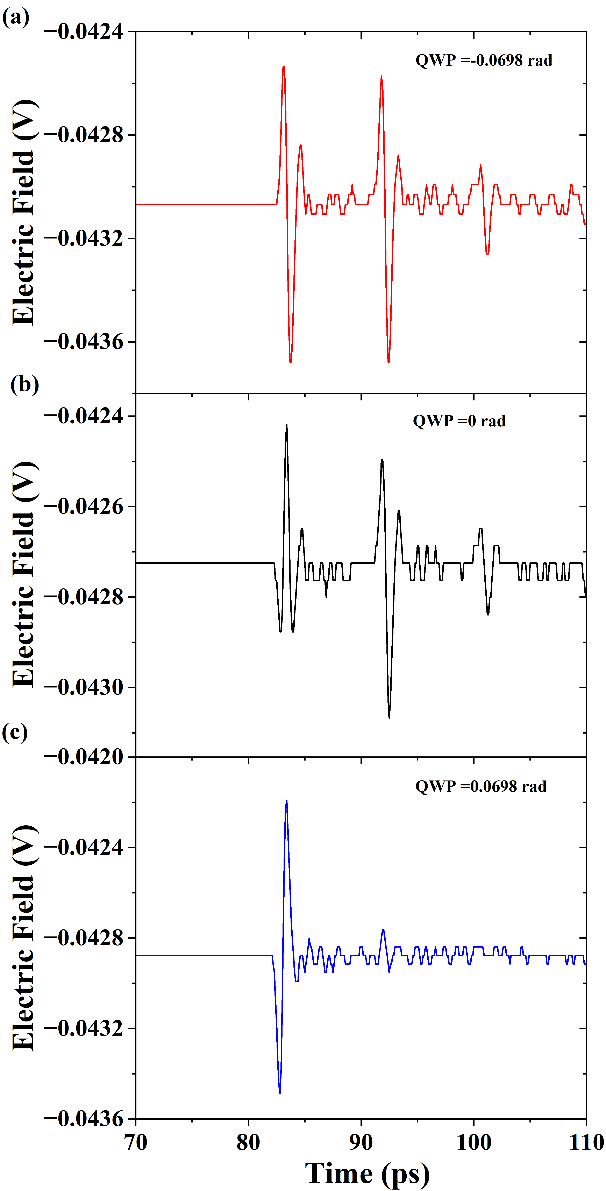


**Figure S1.** Different quarter-wave plate angular positions correspond to THz electric fields characterized by carrier-envelope phases (CEPs). (a) THz time-domain electric field diagram of the QWP rotated -0.0698 rad. (b) THz time-domain electric field diagram of the QWP in the initial position (no rotation). (c) THz time-domain electric field diagram of the QWP rotated 0.0698 rad.

**2. Terahertz phase detection using weak-value amplification with different post-selections**

In addition to Figure 3, we also utilized the terahertz system depicted in Figure 1a with different post-selections to measure the phase shift with κ = ±0.0698 rad in Figure S2, and κ = ±0.0872 rad in Figure S3. Therefore, the results in Figures S2 and S3 will not be discussed further. For κ = ±0.0698 rad, the sensitivity of the phase is calculated$S_{\alpha}={\Delta\left( \left| E_{\alpha}^{+} \right|-\left| E_{\alpha}^{-} \right| \right)}/{\Delta\alpha=0.3175}V/rad$, and the standard deviation of the peak-to-peak value is measured as$\sigma\left| E_{\alpha} \right|=8.71\times{10}^{-5} V$, resulting in a terahertz wave phase resolution of $R_{\alpha}={\sigma\left| E_{\alpha} \right|}/{S_{\alpha}=}2.74\times{10}^{-4} rad$. Similarly, for κ = ±0.0872 rad, the sensitivity of the phase is calculated$S_{\alpha}={\Delta(\left| E_{\alpha}^{+} \right|-\left| E_{\alpha}^{-} \right|)}/{\Delta\alpha=0.3173}V/rad$, and the standard deviation of the peak-to-peak value is measured as $\sigma\left| E_{\alpha} \right|=3.03\times{10}^{-4} V$, resulting in a terahertz wave phase resolution of $R_{\alpha}={\sigma\left| E_{\alpha} \right|}/{S_{\alpha}=}9.56\times{10}^{-4} rad$. These results indicate that the stability of our system ensures that the resolution of different post-selected phases is within 10^-4^ rad.


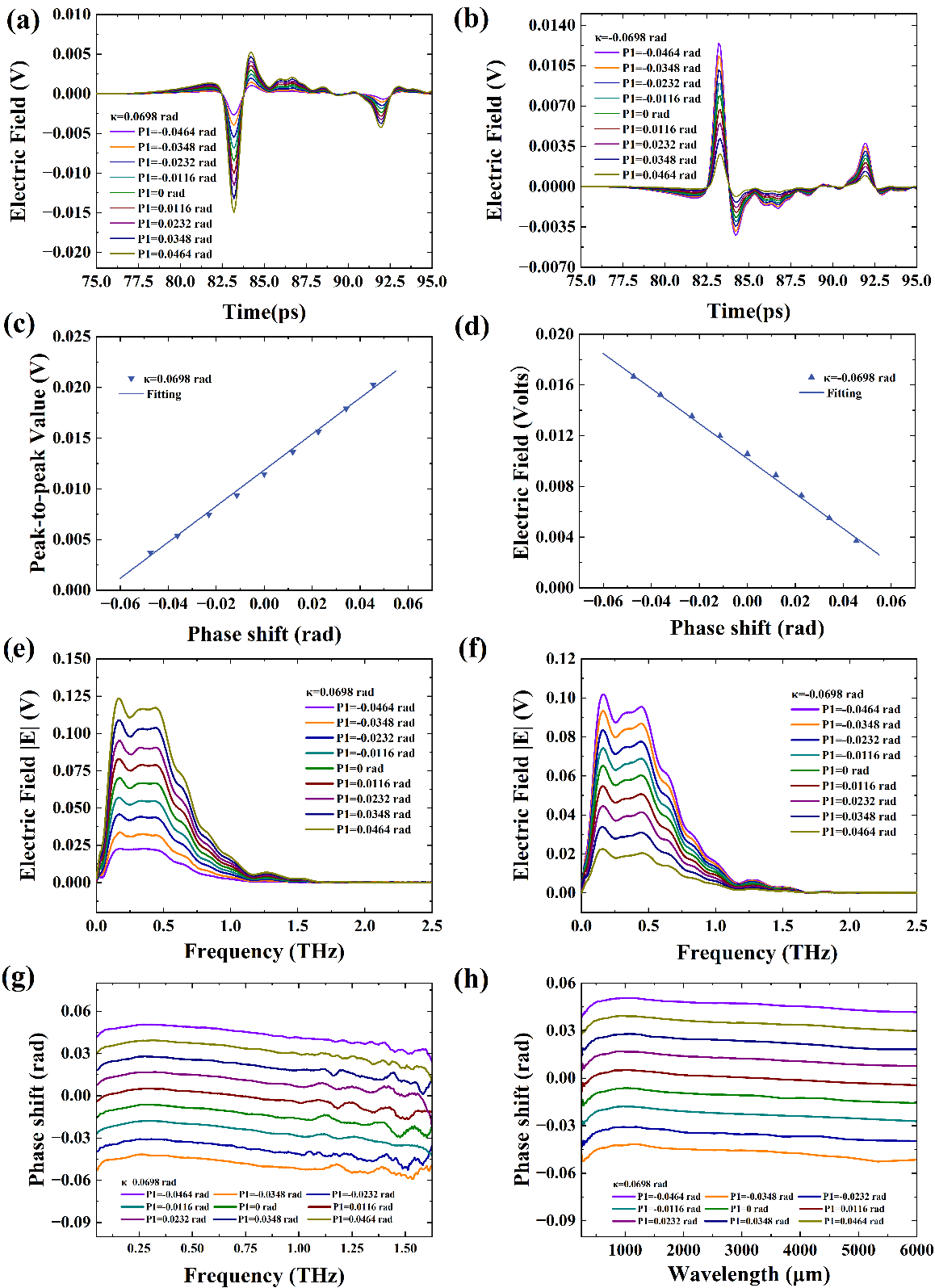


**Figure S2.** Experimental results in time and frequency domains show phase shift measurements with post-selected angle κ = ±0.0698 rad. (a) Electric field with κ =0.0698 rad in time domain. (b) Electric field with κ =-0.0698 rad in time domain. (c) Peak-to-peak signal with κ =0.0698 rad in time domain. (d) Peak-to-peak signal with κ =-0.0698 rad in time domain. (e) Frequency domain spectroscopy with κ = 0.0698 rad. (f) Frequency domain spectroscopy with κ = -0.0698 rad. (g) Function of phase shift α and frequency. (h) Function of phase shift α and wavelength.


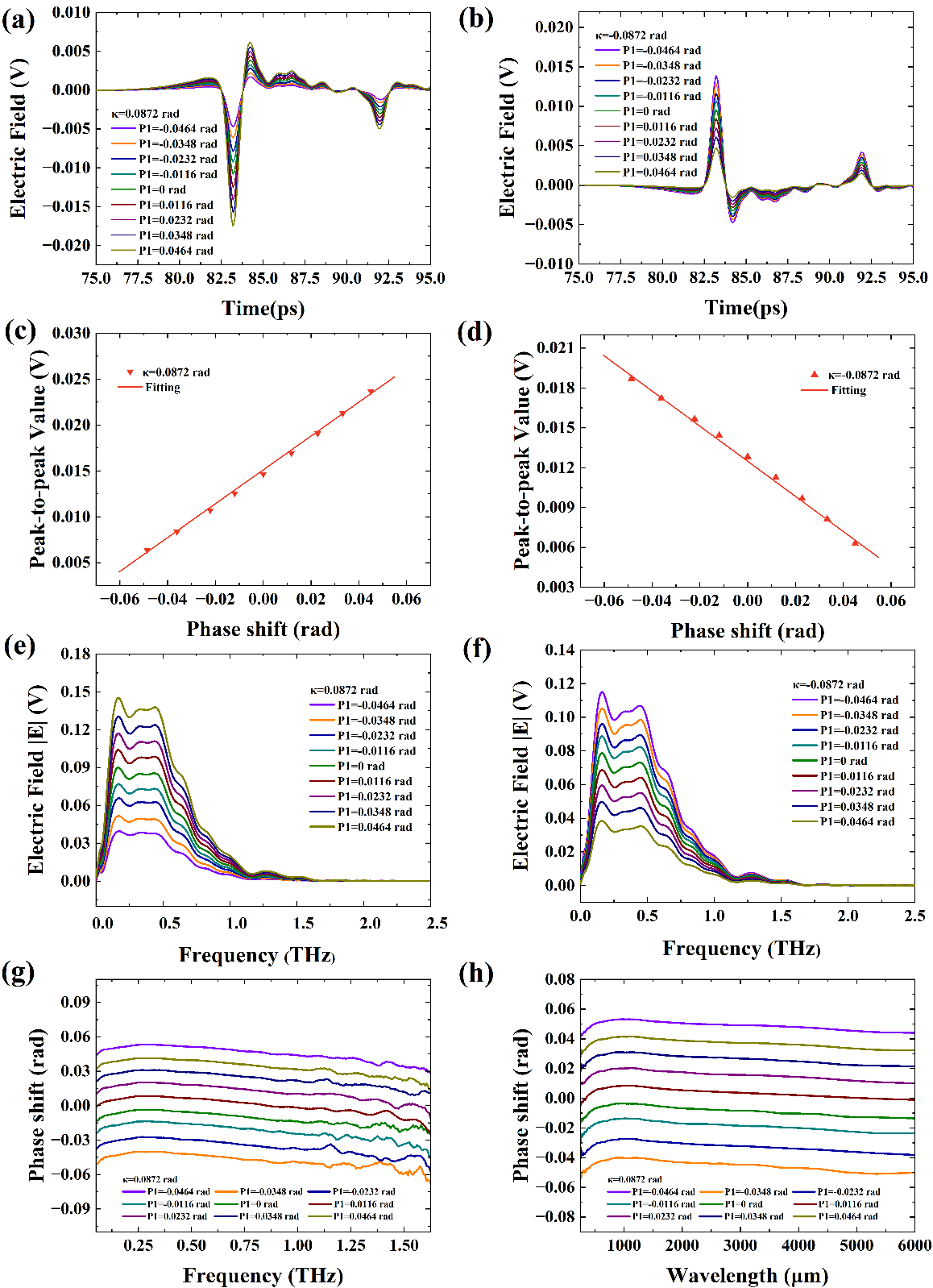


**Figure S3.** Experimental results in time and frequency domains show phase shift measurements with post-selected angle κ = ±0.0872 rad. (a) Electric field with κ =0.0872 rad in time domain. (b) Electric field with κ =-0.0872 rad in time domain. (c) Peak-to-peak signal with κ =0.0872 rad in time domain. (d) Peak-to-peak signal with κ =-0.0872 rad in time domain. (e) Frequency domain spectroscopy with κ = 0.0872 rad. (f) Frequency domain spectroscopy with κ = -0.0872 rad. (g) Function of phase shift α and frequency. (h) Function of phase shift α and wavelength.

**3. Terahertz amplitude detection using weak-value amplification with different post-selections**

In addition to Figure 4, we also utilized the terahertz system depicted in Figure 1b with different post-selections to measure the amplitude shift with κ = ±0.0698 rad in Figure S4, and κ = ±0.0872 rad in Figure S5. Therefore, the results in Figures S4 and S5 will not be discussed further. For χ = ±0.0698 rad, the sensitivity of the amplitude is calculated$S_{\beta}={\Delta(\left| E_{\beta}^{+} \right|-\left| E_{\beta}^{-} \right|)}/{\Delta\beta=0.4102 V/rad}$, and the standard deviation of the peak-to-peak value is measured as $\sigma\left| E_{\beta} \right|=3.35\times{10}^{-4} V$, resulting in a terahertz wave amplitude resolution of $R_{\beta}={\sigma\left| E_{\beta} \right|}/{S_{\beta}=}8.16\times{10}^{-4} rad$. Similarly, for χ = ±0.0872 rad, the sensitivity of the phase is calculated$S_{\beta}={\Delta(\left| E_{\beta}^{+} \right|-\left| E_{\beta}^{-} \right|)}/{\Delta\beta=0.4189}V/rad$, and the standard deviation of the peak-to-peak value is measured as $\sigma\left| E_{\beta} \right|=1.76\times{10}^{-4} V$, resulting in a terahertz wave amplitude resolution of $R_{\beta}={\sigma\left| E_{\beta} \right|}/{S_{\beta}=4.2\times{10}^{-4} rad}$. These results indicate that the stability of our system ensures that the resolution of different post-selected amplitude is within ${10}^{-4} rad$.


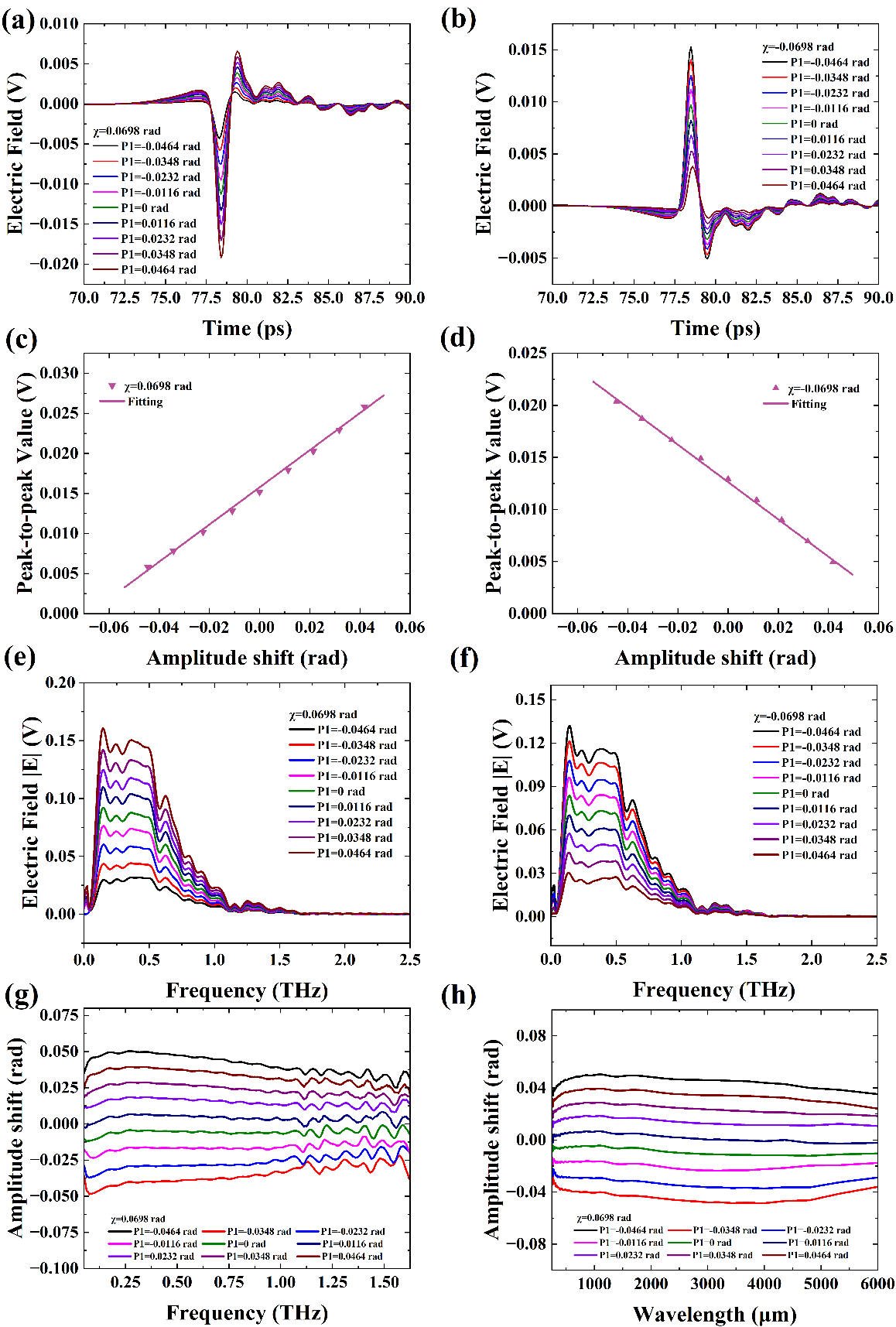


**Figure S4.** Experimental results in time and frequency domains show amplitude shift measurements with post-selected angle χ=±0.0698 rad. (a) Electric field with χ = 0.0698 rad in time domain. (b) Electric field with χ = -0.0698 rad in time domain. (c) Peak-to-peak signal with χ = 0.0698 rad in time domain. (d) Peak-to-peak signal with χ = -0.0698 rad in time domain. (e) Frequency domain spectroscopy with χ = 0.0698 rad. (f) Frequency domain spectroscopy with χ = -0.0698 rad. (g) Function of amplitude shift β and frequency. (h) Function of amplitude shift β and wavelength.


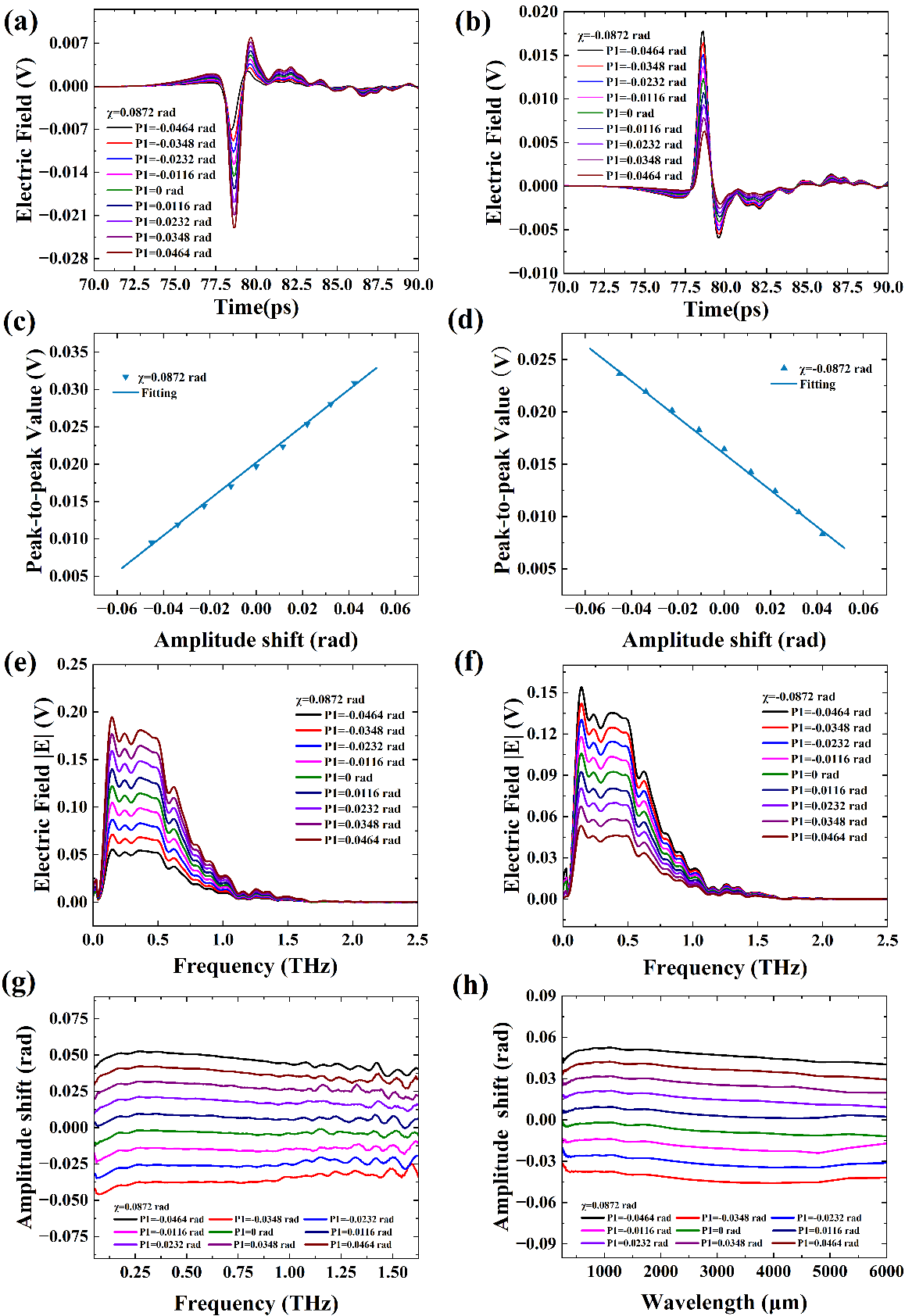


**Figure S5.** Experimental results in time and frequency domains show amplitude shift measurements with post-selected angle χ=±0.0872 rad. (a) Electric field with χ = 0.0872 rad in time domain. (b) Electric field with χ = -0.0872 rad in time domain. (c) Peak-to-peak signal with χ = 0.0872 rad in time domain. (d) Peak-to-peak signal with χ = -0.0872 rad in time domain. (e) Frequency domain spectroscopy with χ = 0.0872 rad. (f) Frequency domain spectroscopy with χ = -0.0872 rad. (g) Function of amplitude shift β and frequency. (h) Function of amplitude shift β and wavelength.
